# Liraglutide dictates macrophage phenotype in apolipoprotein E null mice during early atherosclerosis

**DOI:** 10.1186/s12933-017-0626-3

**Published:** 2017-11-06

**Authors:** Robyn Bruen, Sean Curley, Sarina Kajani, Daniel Crean, Marcella E. O’Reilly, Margaret B. Lucitt, Catherine G. Godson, Fiona C. McGillicuddy, Orina Belton

**Affiliations:** 1 Diabetes Complications Research Centre, School of Biomolecular and Biomedical Science, UCD Conway Institute, University College Dublin, Dublin 4, Ireland; 2 Diabetes Complications Research Centre, School of Medicine, UCD Conway Institute, University College Dublin, Dublin 4, Ireland; 3 Diabetes Complications Research Centre, School of Veterinary Medicine, UCD Conway Institute, University College Dublin, Dublin 4, Ireland; 4 School of Medicine, Department of Pharmacology and Therapeutics, Trinity College Dublin, The University of Dublin, College Green, Dublin 2, Ireland

**Keywords:** MΦ2 macrophages, Monocytes, Anti-inflammatory, Plaque microenvironment, Atherosclerosis

## Abstract

**Background:**

Macrophages play a pivotal role in atherosclerotic plaque development. Recent evidence has suggested the glucagon-like peptide-1 receptor (GLP-1R) agonist, liraglutide, can attenuate pro-inflammatory responses in macrophages. We hypothesized that liraglutide could limit atherosclerosis progression in vivo via modulation of the inflammatory response.

**Methods:**

Human THP-1 macrophages and bone marrow-derived macrophages, from both wild-type C57BL/6 (WT) and apolipoprotein E null mice (ApoE^−/−^) were used to investigate the effect of liraglutide on the inflammatory response in vitro. In parallel, ApoE^−/−^ mice were fed a high-fat (60% calories from fat) high-cholesterol (1%) diet for 8 weeks to induce atherosclerotic disease progression with/without daily 300 μg/kg liraglutide administration for the final 6 weeks. Macrophages were analysed for MΦ1 and MΦ2 macrophage markers by Western blotting, RT-qPCR, ELISA and flow cytometry. Atherosclerotic lesions in aortae from ApoE^−/−^ mice were analysed by en face staining and monocyte and macrophage populations from bone marrow derived cells analysed by flow cytometry.

**Results:**

Liraglutide decreased atherosclerotic lesion formation in ApoE^−/−^ mice coincident with a reduction in pro-inflammatory and increased anti-inflammatory monocyte/macrophage populations in vivo. Liraglutide decreased IL-1beta in MΦ0 THP-1 macrophages and bone marrow-derived macrophages from WT mice and induced a significant increase in the MΦ2 surface marker mannose receptor in both MΦ0 and MΦ2 macrophages. Significant reduction in total lesion development was found with once daily 300 μg/kg liraglutide treatment in ApoE^−/−^ mice. Interestingly, liraglutide inhibited disease progression at the iliac bifurcation suggesting that it retards the initiation and development of disease. These results corresponded to attenuated MΦ1 markers (CCR7, IL-6 and TNF-alpha), augmented MΦ2 cell markers (Arg-1, IL-10 and CD163) and finally decreased MΦ1-like monocytes and macrophages from bone marrow-derived cells.

**Conclusions:**

This data supports a therapeutic role for liraglutide as an atheroprotective agent via modulating macrophage cell fate towards MΦ2 pro-resolving macrophages.

**Electronic supplementary material:**

The online version of this article (10.1186/s12933-017-0626-3) contains supplementary material, which is available to authorized users.

## Introduction

Macrovascular complications of diabetes mellitus (DM), primarily atherosclerosis are the primary cause of morbidity and mortality within affected patients. Approximately 68% of DM patients over the age of 65 die from a cardiovascular related-illness [1]. Framingham data with 20 year follow-up revealed that patients with DM had a 2–3-fold increase in atherosclerotic disease [2]. Similarly, in the Multiple Risk Factor Intervention Trial, males with type 2 DM had a threefold risk of coronary artery disease-related death, compared to non-diabetic controls [3]. Most of the molecular and cellular mediators involved in atherosclerosis have been elucidated [4] and an increasing body of evidence has identified the pivotal role of macrophages in atherosclerotic plaque formation [5]. In addition, diabetes-associated hyperglycemia and oxidative stress contribute to accelerated atherosclerosis by directly effecting monocytes and macrophages [6].

Atherosclerosis is a chronic progressive inflammatory disease and cholesterol metabolism disorder. Monocytes are recruited to, and migrate through the damaged endothelium where they differentiate to macrophages in the intimal layer [7]. “Classically activated” MΦ1 macrophages are pro-atherogenic and differentiate upon exposure to T-helper 1 cytokines, IFN-gamma and IL-1beta; and sustain the ongoing inflammatory response via generation of TNF-alpha and IL-1beta. “Alternatively activated” MΦ2 macrophages are atheroprotective and arise from exposure to T-helper 2 cytokines, IL-4 and IL-13 [8] and promote tissue repair and healing [9].

Interestingly, MΦ1/MΦ2 macrophages show plasticity in response to cues from the microenvironment. MΦ1 and MΦ2 macrophages have a functional role in both obesity and atherosclerosis [10, 11]. Macrophages within atherosclerotic plaques are typically MΦ1 promoting chronic inflammatory responses [8]. However, it has been proposed that atherosclerosis-associated inflammation may not primarily be due to a skewing of the MΦ1:MΦ2 ratio, but also loss of anti-inflammatory MΦ2 macrophages resulting in impaired resolution of inflammation [8, 12]. In the context of atherosclerosis, we have shown that there is an MΦ2 to MΦ1 switch during human plaque progression [13]. In addition we showed that MΦ2 macrophages are localized to stable regions of atherosclerotic plaques and that expression of MΦ2 macrophage cell markers is inversely related to disease progression [13]. Type 2 DM is characterized by a marked reduction in MΦ2 populations and a shift in the ratio between MΦ1 and MΦ2 macrophages is directly related to the development of insulin resistance in adipose tissue [14]. This suggests that skewing macrophage phenotypes is an important mechanism through which DM induces cardiovascular disease (CVD) risk. We propose that therapeutic interventions which induce a MΦ2 switch may protect against diabetes-induced development of CVD.

Since the publication of the “Liraglutide effect and action in diabetes: evaluation of cardiovascular outcome results—a long term evaluation (LEADER) trial”, more emphasis has been placed on the potential protective effects of incretin-based therapies [15]. Liraglutide, a glucagon-like peptide-1 receptor (GLP-1R) agonist currently used for the treatment of obesity-associated type 2 DM, confers minimum risk of hypoglycemia and promotes weight loss [16]. The results of the LEADER trial showed that diabetic patients had a 13% reduction in risk of CV deaths following liraglutide administration [15]. Furthermore, the Evaluation of Lixisenatide in Acute Coronary Syndrome (ELIXA) trial showed that lixisenatide confers a CV benefit rather than an absence of adverse effects [17]. Since increased benefit in CV outcomes with GLP-1R agonists have now been established, investigating the effects of GLP-1R agonists on atherosclerosis-associated inflammation and determining their atheroprotective mechanism is important.

Liraglutide promotes anti-inflammatory responses [18–20], reduces foam cell formation [21, 22], inhibits expression of inflammatory markers and monocyte adhesion and attenuates atherosclerosis in vivo [23, 24]. The aim of our study was to investigate if liraglutide induces an MΦ2 macrophage phenotype and promotes resolution in an in vivo model of early atherosclerosis progression.

## Methods

### Animals and diets

Homozygous ApoE^−/−^ mice (002052; C57BL/6J-ApoE^tm1Unc^; RRID:IMSR_TAC:apoe) were purchased from Charles River Laboratories (Margate, UK) and housed in specific pathogen free conditions in 12 h light and dark cycle. All animal experiments were conducted according to Institutional guidelines and in compliance with the Health Regulatory Products Agency Ireland and Directive 2010/63/EU. Number of experimental animals was based on the sample size calculation (see Additional file 1: Methods). Diets were composed of 10% fat, 0% no added cholesterol (low-fat diet, LFD) or 60% fat with 1% cholesterol (high-fat, high-cholesterol diet, HFHCD). Both diets were sucrose matched (10%) and were supplied by Research Diets (New Brunswick, NJ, USA). Male ApoE^−/−^ mice were randomized at 8 weeks of age to receive LFD or HFHCD for 8 weeks. For the final 6 weeks mice received daily subcutaneous injections of PBS or liraglutide (Victosa, Novo Nordisk, Dublin, Ireland). Liraglutide dose was titrated upwards for the first 10 days (1, 3, 10, 30, 50, 100, 150, 200, 250 and 300 μg/kg) and maintained at 300 μg/kg. Mice were scored, weighed and water intake measured once weekly for the first 2 weeks and daily for the remaining 6 weeks. Food intake was measured weekly. Mice were euthanized by retro-orbital bleed and cervical dislocation and aortae and bones harvested. No adverse events were reported. The unit of analysis was a single animal.

### Peripheral blood mononuclear cell isolation

Peripheral venous blood was collected from healthy volunteers at University College Dublin and Irish Blood Transfusion Service, National Blood Bank, St. James’s Hospital, James’s Street, Dublin 8., post institutional review board ethical approval. Written informed consent was obtained from all volunteers. Peripheral blood mononuclear cells (PBMCs) were isolated from blood as previously described [25]. Briefly, 20 ml of blood was collected, layered onto polymorphoprep solution (Axis-Shield, Dundee, UK) (1:1) and centrifuged for 35 min at 500×*g* and 20 °C. The mononuclear layer was removed, mixed with an equal volume of 0.45% NaCl (Sigma-aldrich, Dorset, UK) and centrifuged for 10 min at 400×*g* and 20 °C. The pellet was re-suspended in 12 ml ice-cold water and 12 ml 1.8% NaCl and centrifuged for 5 min at 300×*g* at 20 °C. Cells were re-suspended in complete medium [RPMI + Glutamax supplemented with 10% FBS and 100U penicillin–streptomycin (Gibco, Thermo Fisher Scientific, Waltham, MA, USA)], counted and seeded at a density of 2.5 × 10^5^ cells/ml.

### Cell line

THP-1 monocytes were seeded at a density of 1 × 10^6^ cells/2 ml medium (ATCC^®^ TIB-202™, Teddington, Middlesex, UK, CLS Cat# 300356/p804_THP-1, RRID:CVCL_0006) and differentiated into MΦ0 cells using 320 nmol/l phorbol 12-myristate 13-acetate (PMA) (Sigma-aldrich, Dorset, UK) for 72 h, rested in complete medium for 24 h and polarized with 100 ng/ml lipopolysaccharide (LPS) (InvivoGen, Toulouse, France) and 20 ng/ml IFN-gamma (R&D Systems, Abingdon, UK) to induce a MΦ1, or 20 ng/ml IL-4 and IL-13 (R&D Systems, Abingdon, UK) for a MΦ2 macrophage phenotype for 48 h. THP-1 MΦ0, MΦ1 and MΦ2 macrophages were treated post-polarization with 250 nmol/l (~ 1 μg/ml) liraglutide for 6 h. mRNA and protein were isolated from cells for RT-qPCR and Western blotting (Additional file 1: Methods) analysis for mannose receptor (MR) expression. Cells tested negative for mycoplasma contamination.

### Bone marrow-derived macrophage (BMDM) culture

Murine BMDMs were taken from C57BL/6 (RRID:MGI:3038854) and ApoE^−/−^ femurs and tibiae and lavaged with complete medium. Cells were cultured in 75% RPMI + Glutamax supplemented with 10% FBS and 1% l-glutamine (Gibco, Thermo Fisher Scientific, Waltham, MA USA) and 25% L929-conditioned medium for 7 days. On day 3 the medium was replaced. On day 7 cells were treated with liraglutide (250 nmol/l for 6 h) or polarized into MΦ1 and MΦ2 macrophages using 100 ng/ml LPS and 20 ng/ml IFN-gamma or 20 ng/ml IL-4 and IL-13 (R&D Systems, Abingdon, UK), respectively for 18 h.

### ELISA

Supernatants were collected from THP-1 and BMDM cells and analyzed by ELISA for human IL-1beta, IL-10, monocyte chemoattractant protein (MCP)-1 and TNF-alpha or murine IL-1beta, IL-10 and IL-6 (eBioscience, Thermo Fisher Scientific, and Biolegend, San Diego, CA, USA) as per the Manufacturer’s instructions.

### Western blotting

THP-1 cells were harvested for protein. 20 μg of protein was quantified by a Bradford assay (Bio-rad, Fannin Ltd., Dublin, Ireland) as per the manufacturers instructions. The protein was run using 10% sodium dodecyl sulfate–polyacrylamide gel electrophoresis (Additional file 1: Table S1) at 90 V for 10 min and 120 V for a further 95 min. The proteins were then transferred onto nitrocellulose membrane (VWR, Dublin, Ireland) at 115 V for 1.5 h. The membrane was blocked in 5% non-fat skimmed milk for 1 h at room temperature and probed at 4 °C overnight for MR, 1:500, Signal transducer and activator of transcription (STAT)3 1:1000, STAT1 1:1000, phospho (p)-STAT1 1:1000 (Cell Signalling Technology, Dublin, Ireland), GLP-1R 1:1000 or beta-actin (Santa Cruz Biotechnology, Heidelberg, Germany) was used as the loading control at 1:1000. Membranes were then probed with anti-rabbit 1:1000 (MR, STAT3, STAT1, p-STAT1 and GLP-1R) or anti-mouse 1:2000 (beta-actin only) as a secondary antibody for 1 h at room temperature and developed in Pierce™ ECL Western blotting substrate (Thermo Fisher Scientific, Dublin, Ireland).

### Quantitative RT-PCR

RNA from cells was extracted using the Qiagen RNeasy kit (Qiagen, Manchester, UK) as per Manufacturer’s instructions. RNA quantity and quality was determined using the NanoDrop™ 2000 (Life Technologies Ltd., Paisley, UK). mRNA was converted to cDNA and analyzed by RT-qPCR on ABI PRISM 7900HT (Applied Biosystems, Life Technologies, Ireland) using a TaqMan hydrolysis probe for mannose receptor C-type 1 (*Mrc1,* HGNC ID:7228), *Il*-*10* (HGNC ID: 601), arginase-1 (*Arg1,* HGNC ID: 663) and 18S ribosomal RNA (HGNC ID: 1383) as a reference gene (Applied Biosystems, Life Technologies, Dublin, Ireland) and SYBR^®^ Green primers listed in Additional file 1: Table S2 (Eurofins, MWG Operon, Ebersberg, Germany).

### En face analysis

Aortae were harvested from ApoE^−/−^ mice and fixed in 10% formalin (DiaPath, Martinengo BG, Italy). Adventitial fat tissue was removed and the aortae opened longitudinally, rinsed in 70% ethanol for 1 min, stained in Sudan IV (35% ethanol/50% acetone) (Sigma-Aldrich, Dorset, UK) for 15 min with continuous agitation, destained in 80% ethanol for 2 min with agitation and rinsed until solution was clear in deionized water. Aortae were pinned onto black wax and imaged using a 4× magnification. Photoshop (Adobe Systems Inc., San Jose, CA, USA) was used to stitch images together and lesions were quantified using ImageJ (NIH, Bethesda, MD, USA) software. Percentage atherosclerotic lesion area was calculated as a ratio of the total aorta area to the lesion area.

### Flow cytometry

BMDMs (2 × 10^6^) from mice fed LFD or HFHCD (with/without liraglutide treatment) were labelled after 7 days in culture with anti-mouse monocyte and macrophage specific markers (BD Biosciences, Oxford, UK) (Table 1). Antibodies were combined to make a master mix using brilliant buffer (BD Biosciences, Oxford, UK) and PBS. Cells were stained with the antibody mix for 30 min at room temperature in the dark, washed and re-suspended in PBS before running on the Beckman Coulter CyAn Advanced Digital Processing (Beckman Coulter, Brea, CA, USA). Cells were also stained with a combination of all antibodies except one for fluorescence minus one controls for gating controls and amine C reactive positive and negative beads were stained with one antibody for single stained controls required for compensation. Flow cytometry standard (FCS) files were analyzed using FlowLogic software (Miltenyi Biotec Ltd., Surrey, UK).

**Table 1:** Antibodies for flow cytometry

Marker	Flurochrome	Isotype	Clone
CD45	Brilliant ™ Blue (BB)515	rat IgG2b, κ	30-F11
CD11c	Phycoerythrin–cyanine 7 (PE-Cy7)	hamster IgG1, λ1	HL3
Ly-6C	Allophycocyanin (APC)	rat IgM, κ	AL-21
F4/80	Brilliant violet™ (BV)421	rat IgG2a, κ	T45-2342
CD11b	BV510	rat IgG2b, κ	M1/70

### Statistical analysis

Results were analysed using GraphPad Prism 5.0c (GraphPad Software Inc., La Jolla, CA, USA) and are expressed as mean (SEM). Statistical comparisons for all THP-1 and BMDM experiments were made by paired (Friedman) and ApoE^−/−^ in vivo experiments were analyzed by unpaired (Kruskal–Wallis) non-parametric ANOVAs followed by Dunn’s multiple comparison post-tests comparing all columns. Student’s paired (Wilcoxin-matched pairs signed rank) and unpaired (Mann–Whitney) non-parametric t tests were used to compare all columns of interest for in vitro or in vivo work involving RT-qPCR, flow cytometry and en face lesion quantification data. Statistical significance was considered when *p < 0.05, **p < 0.01 and ***p < 0.001.

## Results

### Liraglutide inhibits MCP-1 in human PBMCs

**Fig. 1:**
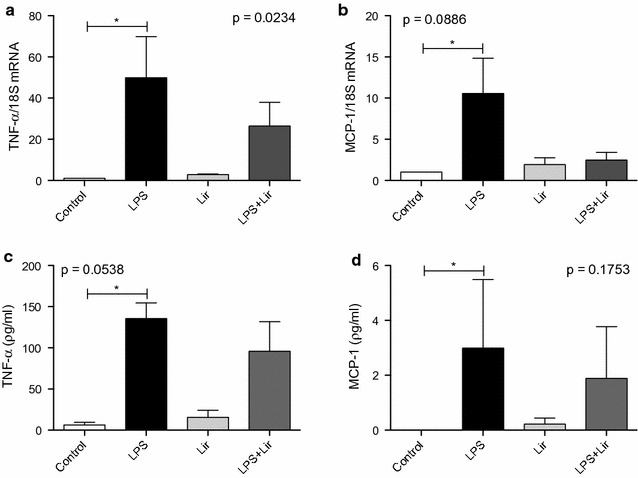
Human PBMCs treated with liraglutide. Human PBMCs were co-treated with LPS 1 μg/ml and liraglutide 1 μmol/l for 2 h. Cells were analyzed for **a** and **c** TNF-alpha and **b** and **d** MCP-1 by **a**, **b** RT-qPCR and **c**, **d** ELISA. Error bars are representative of cells from three healthy volunteers (n = 3). Statistical analysis was carried out performing a Kruskal–Wallis test followed by Dunn’s multiple comparison post-test. Statistical significance was considered when *p < 0.05. Non-significance (NS) was considered when p > 0.05. Capped lines indicate comparisons made between groups which are of statistical significance

MCP-1 mediates rolling and transmigration of monocytes in early atheroscleortic lesion formation [26]. To investigate if liraglutide impacts on MCP-1, human PBMCs were isolated from healthy volunteers and co-treated with 1 μmol/l liraglutide and 1 μg/ml LPS for 2 h and cells analyzed by RT-qPCR and ELISA for TNF-alpha and MCP-1 expression and secretion. Liraglutide attenuated TNF-alpha expression and significantly inhibited MCP-1 expression (Fig. 1a, b). Similar trends were also observed for protein secretion (Fig. 1c, d). This suggests liraglutide impacts on the inflammatory state of monocytes and may function as an anti-inflammatory mediator.

### Liraglutide enhances a MΦ2 phenotype in THP-1 macrophages

**Fig. 2:**
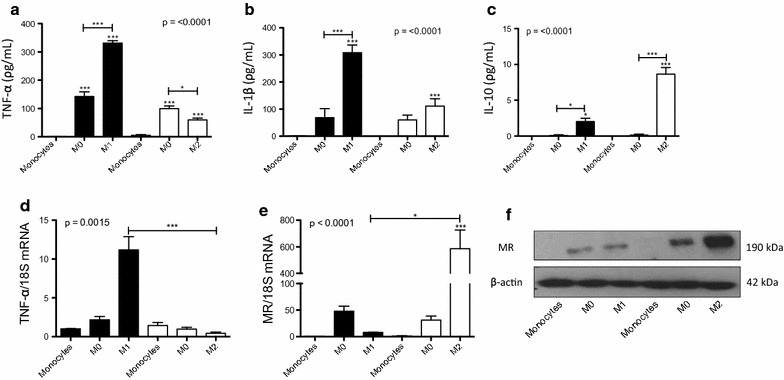
Polarization of THP-1 monocytes to macrophages. THP-1 monocytes were differentiated into MΦ0 macrophages with 320 nmol/l PMA and further polarized to MΦ1 (100 ng/ml LPS and 20 ng/ml IFN-gamma) and MΦ2 (20 ng/ml IL-4 and 20 ng/ml IL-13) macrophages. Polarization was confirmed by analysing cell supernatants for **a** TNF-alpha **b** IL-1beta and **c** IL-10. Cells were analysed for **d** TNF-alpha and **e** mannose receptor (MR) by RT-qPCR and **f** MR by Western blotting. Error bars are representative of three independent experiments (n = 3) each carried out with two replicates. Statistical analysis was carried out performing a Friedman test followed by Dunn’s multiple comparison post-test. *p < 0.05, **p < 0.01 and ***p < 0.001 were considered statistically significant. p > 0.05 was considered NS. Stars above the columns represent comparisons made against monocyte controls while capped lines indicate comparisons made between other groups. A representative Western blot is shown with beta-actin used as a loading control

To investigate if liraglutide impacts on macrophage phenotype, THP-1 monocytes were polarized into MΦ0, MΦ1 and MΦ2 macrophages. Supernatants were analyzed by ELISA for the MΦ1 cytokines, IL-1beta and TNF-alpha, and MΦ2 cytokine IL-10 to confirm polarization (Fig. 2a–c). All pro-inflammatory and anti-inflammatory cytokines tested were significantly increased in MΦ1 or MΦ2 macrophages respectively compared to the MΦ0 control. Although it appears MΦ2’s secrete more IL-1beta and MΦ1’s secrete more IL-10, it is polarized macrophages that secrete more cytokines than naïve macrophages and monocytes. The MΦ2 macrophages do not secrete more IL-1beta then the MΦ1 macrophages and similarly the MΦ1 macrophages do not secrete more IL-10 than the MΦ2 macrophages. Polarizing MΦ1 macrophages requires LPS which in turn activates NF-κB which is known to activate STAT3 and IL-10 signalling [27]. THP-1 macrophage polarization was also confirmed by RT-qPCR analysis of TNF-alpha, an MΦ1 cytokine, and MR, an MΦ2 surface marker (Fig. 2d, e). Western blotting confirmed an increase in MR expression in MΦ2 macrophages (Fig. 2f). This validates THP-1 cells as a model where the effects of liraglutide on the MΦ1/MΦ2 macrophage paradigm can be investigated.

**Fig. 3:**
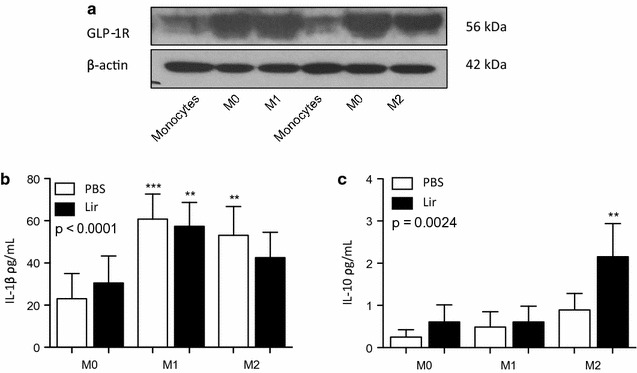
THP-1 macrophages treated with liraglutide. The presence of **a** GLP-1R was detected by Western blotting with beta-actin used as a loading control in THP-1 monocytes and macrophages. A representative Western blot is shown. THP-1 cells were differentiated into MΦ1 (100 ng/ml LPS and 20 ng/ml IFN-gamma) and MΦ2 (20 ng/ml IL-4 and 20 ng/ml IL-13) macrophages. Polarized THP-1 macrophages were treated with 1 μg/ml liraglutide for 6 h and supernatants were collected and analyzed by ELISA for **b** IL-1beta and **c** IL-10. Error bars are representative of three independent experiments (n = 3) each carried out with two replicates. Statistical analysis was carried out performing a Friedman test followed by Dunn’s multiple comparison post-test. **p < 0.01 was considered statistically significant. p > 0.05 was NS. Stars above the columns represent comparisons made against the MΦ0 PBS control

To investigate whether liraglutide acts via a receptor-dependent or receptor-independent mechanism the precsence of the receptor was confirmed by Western blot (Fig. 3a). To elucidate if liraglutide impacts on macrophage cell fate, MΦ1 and MΦ2 macrophages were treated with liraglutide for 6 h, to investigate the effect of the GLP-1R agonist on macrophage phenotype and function (Fig. 3b, c). The dose of liraglutide was based on previous studies by Hogan et al. who treated macrophages with 1 μg/ml (~ 250 nM) liraglutide [28]. Although there was no effect on IL-1beta secretion, liraglutide increased IL-10 secretion in MΦ2 macrophages Furthermore, analysis of expression of the STAT proteins, STAT1 and STAT3, showed that there was a modest decrease in p-STAT1/STAT1 protein, which is involved in pro-inflammatory signalling pathways (Additional file 2: Figure S1). This suggests that liraglutide may enhance an MΦ2 macrophage phenotype necessary for resolution of inflammation via a receptor-dependent mechanism.

### Liraglutide attenuates MΦ1 and promotes MΦ2 phenotypes in WT BMDMs

Although liraglutide enhanced IL-10 secretion from THP-1 MΦ2 macrophages, very low levels were secreted somewhat limiting its use as a model to explore monocyte/macrophage function. Therefore, we employed BMDMs as a more tractable model to test the effect of liraglutide in vitro before investigating the effect of liraglutide in vivo. Bone marrow cells were taken from WT mice and differentiated into MΦ0 macrophages. On day 7 macrophages were polarized into MΦ1 and MΦ2 macrophages. Polarization was confirmed by RT-qPCR for the MΦ1 and MΦ2 markers, TNF-alpha and MR, respectively. There was a significant increase in TNF-alpha in MΦ1 macrophages compared to MΦ0 (20.02 ± 8.45 vs. 4.44 ± 3.03, **p < 0.01) and MΦ2 (20.02 ± 8.45 vs. 1.48 ± 0.68, *p < 0.05) macrophages (Additional file 3: Figure S2a). Furthermore, MR expression was significantly increased in MΦ2 macrophages compared to MΦ0 macrophages (13.67 ± 2.81 vs. 1.08 ± 0.19, *p < 0.05) and MΦ1 (13.67 ± 2.81 vs. 0.73 ± 0.47, *p < 0.05) (Additional file 3: Figure S2b).

**Fig. 4:**
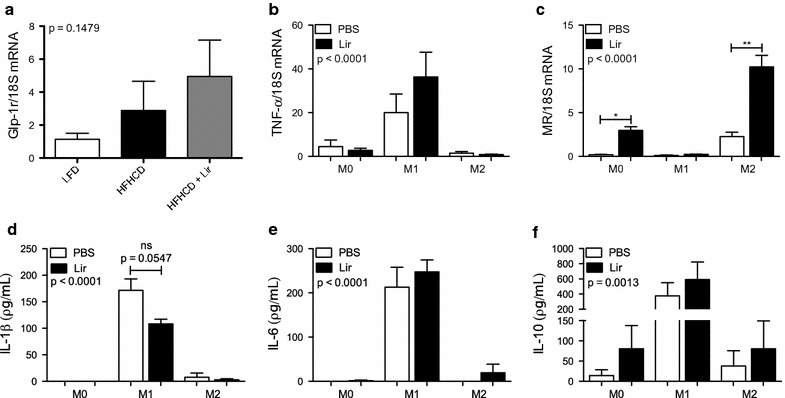
Treatment of MΦ0 WT BMDMs with liraglutide. BMDMs were taken from WT mice and differentiated into MΦ0 macrophages using 25% L929 conditioned medium. MΦ0 macrophages were treated with 1 μg/ml liraglutide ex vivo for 6 h and polarized into MΦ1 (100 ng/ml LPS and 20 ng/ml IFN-gamma) and MΦ2 (20 ng/ml IL-4 and 20 ng/ml IL-13) macrophages for 18 h. Supernatants were analysed by ELISA for **a** IL-1beta **b** IL-6 and **c** IL-10. Cells were analysed by RT-qPCR for **d** TNF-alpha and **e** MR. Error bars are representative of **a**–**c** four mice (n = 4), **d**, **e** three mice (n = 3) each carried out with two replicates. Statistical analysis was carried out performing a Friedman test followed by Dunn’s multiple comparison post-test. *p < 0.05 and **p < 0.01 were considered statistically significant. p > 0.05 was NS. Capped lines indicate comparisons made between groups

On day 7 of culture WT MΦ0 macrophages were pre-treated with liraglutide ex vivo for 6 h and subsequently polarized into MΦ1 and MΦ2 macrophages. Liraglutide-treated MΦ1 macrophages displayed an attenuated MΦ1 phenotype as determined by a trend towards a reduction in IL-1beta secretion (Fig. 4a). However, liraglutide increased MΦ2-like properties (Fig. 4c, d) with a significant increase in MR mRNA expression in both MΦ0 (0.19 ± 0.044 vs. 2.95 ± 0.44, *p < 0.05) and MΦ2 macrophages (2.25 ± 0.51 vs. 10.23 ± 1.31, **p < 0.01) (Fig. 4e) compared to PBS controls. From our data BMDMs are not a good model to examine cytokine secretion but are appropriate for macrophage phenotypic analysis. Thus it was feasible to hypothesize that liraglutide may impact on atherosclerosis development via skewing macrophage populations towards an MΦ2 phenotype. To address this we employed an in vivo model of early atherosclerosis development.

### Liraglutide inhibits atherosclerotic lesion development in ApoE^−/−^ mice

To identify the optimal time point for administration of liraglutide to impact on early atherosclerosis a feeding study was carried out where ApoE^−/−^ mice were fed a HFHCD for 4 or 8 weeks. En face analysis of aortae at 4 weeks resulted in very low levels of plaque burden compared to those fed the same diet for 8 weeks (4 weeks 0.79 ± 0.07% vs. 8 weeks 2.03 ± 0.18%, ***p < 0.001) (Additional file 4: Figure S3a, b). In the aortic arch and iliac bifurcation, there was more lesion formation at 8 weeks, consistent with accelerated lesion formation at branch points in the vessel (Additional file 4: Figure S3c, f). This confirms that 8 weeks HFHCD feeding is the optimal time to investigate the effect of liraglutide on atherosclerosis progression.

**Fig. 5:**
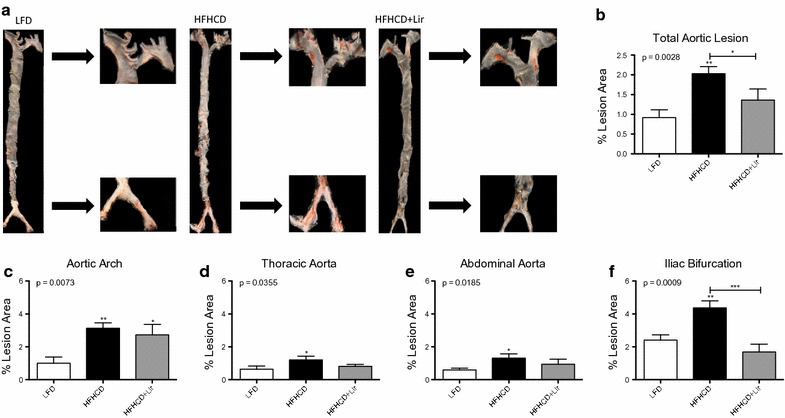
Quantification of atherosclerotic lesions from aortas of ApoE^−/−^ mice. Aortas were harvested from ApoE^−/−^ mice fed a LFD or HFHCD for 8 weeks while receiving daily subcutaneous injections of PBS or liraglutide from weeks 2 to 8. **a** Representative aorta for each treatment group with en face staining. Percent lesion areas were quantified using ImageJ for **b** total aorta, **c** aortic arch, **d** thoracic aorta, **e** abdominal aorta and **f** iliac bifurcation. Error bars are representative of 10 aortas per group (n = 10). Statistical analysis was performed carrying out Mann–Whitney t tests comparing all groups. Statistical significance was considered when *p < 0.05, **p < 0.01 and ***p < 0.001. p > 0.05 was considered NS. Stars above the columns represent comparisons made against the LFD control while capped lines indicate comparisons made between other groups

Liraglutide is administered clinically as a subcutaneous injection at 1.8 mg daily. To minimize adverse effects associated with liraglutide administration we established a model to mimic human regimen on atherosclerosis in vivo. ApoE^−/−^ mice were fed a HFHCD for 2 weeks to induce a dysregulated metabolism. Mice were then administered liraglutide for a total of 6 weeks. The dose of liraglutide was specifically designed to have minimal impact on weight, to facilitate us to investigate weight-independent effects. Atherosclerotic lesion development was analysed by en face staining of the aortae (Fig. 5a). Overall total lesion area was significantly reduced with liraglutide treatment compared to the HFHCD control (2.03 ± 0.18% vs. 1.36 ± 0.28%, *p < 0.05) (Fig. 5b). Interestingly, although there was no difference in lesion development in the aortic arch, thoracic aorta or abdominal aorta (Fig. 5c, d) a significant reduction in lesion burden was evident at the iliac bifurcation of the aorta with liraglutide (3.37 ± 0.43% vs. 1.69 ± 0.47%, **p < 0.01) (Fig. 5f). This halted progression correlated with weights and cholesterol levels of ApoE^−/−^ mice (Additional file 5: Figure S4, Additional file 6: Figure S5) but was independent of food, calorie and water intake with no positive effect on glucose levels (Additional file 7: Figure S6, Additional file 8: Figure S7, Additional file 9: Figure S8).

### Liraglutide promotes MΦ2 macrophage phenotypes in ApoE^−/−^ BMDMs

**Fig. 6:**
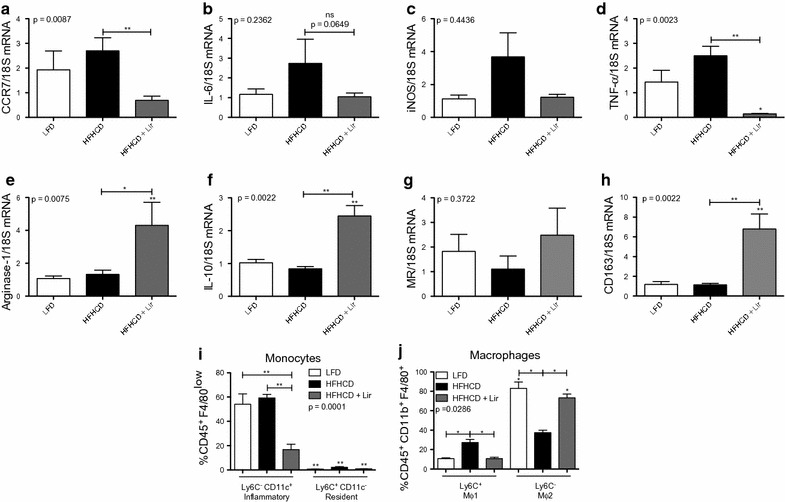
MΦ0 BMDMs from in vivo liraglutide-treated ApoE^−/−^ mice. BMDMs were taken from ApoE^−/−^ mice treated with liraglutide in vivo. BMDMs were differentiated into M0 macrophages and analysed for **a** CCR7, **b** IL-6, **c** iNOS, **d** TNF-alpha, **e** Arg-1, **f** IL-10, **g** MR and **h** CD163 by RT-qPCR with 18SrRNA as the reference gene. BMDMs were also analysed by flow cytometry for **i** monocyte and **j** macrophage populations using antibodies specific against CD45, CD11b, CD11c, F4/80 and Ly-6C. Error bars are representative of 6 mice per group (n = 6) for RT-qPCR and 5 mice per group (n = 5) for monocytes and 4 mice per group (n = 4) for macrophage flow cytometry analysis. Statistical analysis was performed carrying out Mann–Whitney t tests comparing all groups. Statistical significance was considered when *p < 0.05, **p < 0.01 and ***p < 0.001. p > 0.05 was considered NS. Stars above the columns represent comparisons made against the LFD control while capped lines indicate comparisons made between other groups

As shown above liraglutide modulates macrophage phenotypes in vitro. To examine this in vivo, ApoE^−/−^ mice were challenged with a LFD or HFHCD for 8 weeks, receiving injections of PBS or liraglutide for the last 6 weeks. Bone marrow cells were isolated from ApoE^−/−^ mice treated in vivo with liraglutide and differentiated for 7 days into MΦ0 macrophages and characterized by RT-qPCR for MΦ1 (CCR7, iNOS, TNF-alpha and IL-6) (Fig. 6a–d) and MΦ2 (Arg-1, IL-10, MR and CD163) markers (Fig. 6e–h). MΦ1 markers CCR7, TNF-alpha and IL-6 expression was significantly decreased in BMDMs from liraglutide-treated mice. Importantly, in HFHCD liraglutide-treated mice there was a significant increase in expression of MΦ2 macrophage markers specifically, Arg-1 (1.33 ± 0.25 vs. 4.31 ± 1.41, *p < 0.05), IL-10 (0.84 ± 0.07 vs. 2.46 ± 0.32, **p < 0.01) and CD163 (1.14 ± 0.16 vs. 6.80 ± 1.53, **p < 0.01) compared to BMDMs from HFHCD mice. This suggests that liraglutide promotes a MΦ2 macrophage phenotype to inhibit development of atherosclerosis in vivo.

To further validate our hypothesis we employed flow cytometry to analyse the monocyte and macrophage populations from bone marrow differentiated cells in response to liraglutide (gating strategy shown in Additional file 10: Figure S9). On day 7 of culture, suspension cells represented the monocyte population (Fig. 6i) and adherent cells represented the macrophage population (Fig. 6j). The inflammatory MΦ1-like monocyte population was identified by CD45^+^F4/80^lo^Ly-6C^−^CD11c^+^, in contrast to the resident MΦ2-like monocytes identified by CD45^+^F4/80^lo^Ly-6C^+^CD11c^−^. There was a significant decrease in inflammatory monocyte populations following liraglutide treatment (16.79 ± 4.47%) compared to both the LFD (54.01 ± 8.55% **p < 0.01) and HFHCD controls (**p < 0.01) (Fig. 6d), suggesting that liraglutide reduces the inflammatory monocyte cell population in vivo. Macrophage populations were gated on CD45^+^CD11b^+^F4/80^+^Ly-6C^+^ or CD45^+^CD11b^+^F4/80^+^Ly-6C^−^ for MΦ1 and MΦ2, respectively. Liraglutide attenuated the polarization of MΦ1 macrophages in vivo (27.28 ± 3.25% vs. 10.75 ± 1.40%, *p < 0.05) and importantly promoted the differentiation of BMDMs towards MΦ2 macrophages (37.45 ± 2.49% vs. 73.36 ± 3.84%, *p < 0.05). In addition, a trend towards increased MΦ2 macrophages in the epididymal adipose tissue (EAT) was also observed (Additional file 11: Figure S10). These data suggest liraglutide can drive macrophages towards a pro-resolving phenotype thus inhibiting development of atherosclerosis.

## Discussion

The developing atherosclerotic lesion induces a unique microenvironment consisting of immune cells that shift the normal balance towards a pro-inflammatory state. Determining if pharmacological interventions can limit progression or promote resolution of the disease by impacting on macrophage phenotype is important for improved treatment of DM macrovascular complications [29]. The work described here shows that the GLP-1R agonist, liraglutide, has a direct effect on macrophage phenotype in early atherosclerosis resulting in inhibition of lesion formation in vivo.

To date in vivo studies which have examined the atheroprotective effect of GLP-1 receptor agonists have yielded conflicting results. Previous studies have examined the effect of GLP-1R agonists exendin-4 [30] and taspoglutide [31] in vivo. Whilst high doses of exendin-4 (24 nmol kg^−1^day^−1^) for 4 weeks suppressed atherosclerosis in ApoE^−/−^ mice fed a standard diet [30] monthly doses of 0.4 mg taspoglutide for 12 weeks did not reduce plaque area in hyperglycemic ApoE^−/−^ mice fed a 45% fat diet [31]. These results highlight the differences between exendin-based therapies and human GLP-1 analogues (taspoglutide) and the effect hyperglycemia and high fat diets have on responses to incretins. Previous studies have examined the effect of liraglutide on progression of atherosclerotic disease [24, 32]. ApoE^−/−^ mice given 300 μg/kg liraglutide twice daily for 4 weeks, receiving a 22% fat and 0.15% cholesterol diet, had increased plaque stability although there was little effect on lesion area [32]. A more recent study in LDL receptor null mice fed a 4.25% fat diet containing 0.3% cholesterol showed liraglutide at 1000 μg/kg daily for 13 weeks following a nephrectomy had attenuated atherosclerosis [23]. Other in vivo studies have shown liraglutide is cardioprotective in an ischemic/reperfusion injured hearts model by preserving physiological levels of calcium [33] and in a doxorubicin-induced cardiotoxicity model [34]. In our study ApoE^−/−^ mice received a single daily dose of 300 μg/kg liraglutide for 6 weeks resulting in significant reduction of lesion progression at the iliac bifurcation of the aorta. Previous in vivo studies administered liraglutide for 4 weeks [30–32, 35]. 300 μg/kg liraglutide has previously shown to have little effect on weight loss [24, 36] meaning the atheroprotective effects we report are weight-independent. The equivalent dose of liraglutide given subcutaneously in a clinical setting is 1.8 mg/kg, as opposed to 3 mg/kg used to treat obesity [37]. A unique aspect of our model is the high-fat high-cholesterol diet used. It has been extensively documented that ApoE^−/−^ mice on a high-cholesterol diet are more susceptible to rapidly developing atherosclerotic lesions compared mice on a high-fat diet alone [38]. An obesogenic high-fat diet was used in this study to better mirror the current target population of liraglutide. Faber et al. investigated the effect of liraglutide treatment in patients and found no significant results in microvascular function after 10 weeks [39] compared to Rizzo et al. who found significant reduction in waist circumference and intima-media thickness in carotid arteries in patients after an 18 month follow up [40]. The main differences in these studies where the combination of drugs administered to patients and the length of treatment. Rizzo et al. used liraglutide in combination with metformin in contrast to Faber et al. who only gave liraglutide. Both studies used a slightly lower dose of 1.2 mg/kg compared to the standard dose of 1.8 mg/kg. Longer administration had the most profound effect. Although liraglutide had significant effects on intima-media thickness in carotid arteries in patients [40], it had no significant effect in systolic function or exercise capacity [41]. Therefore our data suggests that liraglutide may elicit a similar effect in patients with early stage atherosclerosis or at high risk of atherosclerosis development. Although results were variable in patients with established disease, our work warrants further investigation into translational research.

Previous studies have examined the potential mechanism through which GLP-1R agonists confer atheroprotection. It has been shown that liraglutide reduces monocyte adherence to human aortic endothelial cells in response to TNF-alpha and LPS stimulation [42]. Of relevance to our study, is that exenatide induces MΦ2 macrophage polarization via signal transducer and activator of transcription three in human monocyte derived macrophages [43]. However, few studies have addressed the effect of liraglutide on the inflammatory status of monocytes and macrophages in vivo in the context of atherosclerosis. Most recently Vinué et al. reported that lixisenatide decreases atherosclerosis in insulin resistant mice by reprogramming macrophages towards an MΦ2 phenotype [35]. Indeed, our initial approach using THP-1 monocytes suggested that liraglutide influences monocyte/macrophage cell fate. Liraglutide attenuated IL-1beta in MΦ0 macrophages and showed a trend towards elevated secretion of IL-10 from both MΦ0 and MΦ2 macrophages. Indeed, similar results have been reported by others which showed decreased IL-1beta in MΦ1 THP-1 macrophages and increased IL-10 secretion from MΦ2 THP-1 macrophages [28]. To date there is limited data on the effect of liraglutide on BMDM phenotypes or cell fate in vivo. This is important as we and others have shown that inhibition and regression of atherosclerosis is associated with a phenotypic MΦ1 to MΦ2 macrophage switch [13, 44, 45]. To address this we examined the effect of ex vivo liraglutide treatment on pro-and anti-inflammatory cytokine generation and on expression of macrophage phenotypic markers TNF-alpha (MΦ1) and MR (MΦ2) in BMDMs from WT mice. Similar to what we observed in THP-1 cells, we found a trend towards reduced IL-1beta and enhanced IL-10 secretion in MΦ0, MΦ1 and MΦ2 WT BMDMs following ex vivo liraglutide treatment. This is in keeping with what has been observed with exenatide treatment in human monocyte derived macrophages [43]. This suggests liraglutide acts as an immune modulator that can alter macrophage phenotype. EAT is the gonadal white fat found in male rodents and expands significantly when these mice are fed a high-fat diet [46]. To confirm a role for liraglutide in inducing a pro-resolving macrophage phenotype we examined macrophage infiltration in EAT from ApoE^−/−^ mice fed a HFHCD. We showed reduced macrophage infiltration and a trend towards increased MΦ2 macrophage polarization in EAT (Additional file 11: Figure S10).

To address if liraglutide impacts on macrophage phenotype in disease we examined the effect of in vivo liraglutide treatment on monocyte/macrophage phenotype in the ApoE^−/−^ mice. Animals were administered a HFHCD for 2 weeks to induce a state of metabolic dysfunction which was confirmed with weight gain (Additional file 5: Figure S4), following administration of liraglutide in the presence of the HFHCD for a further 6 weeks. After a total of 8 weeks of diet challenge both the monocyte and macrophage populations were quantified. This is the first study to directly examine alteration in both monocyte and macrophage populations in vivo in response to liraglutide in the context of atherosclerosis. Pro-inflammatory and anti-inflammatory monocyte populations in mice are characterised by CD45^+^F4/80^lo^Ly-6C^hi^CD11c^−^ and CD45^+^F4/80^lo^Ly-6C^lo^CD11c^+^ respectively [47]. Inflammatory monocytes are abundant in ApoE^−/−^ mice where they adhere to the endothelium, infiltrate, differentiate to macrophages and contribute to disease progression [48]. However, the specific role of pro-resolving monocytes in atherosclerosis is less clear [48, 49]. We found a significant change in MΦ1-like characterized monocytes, where these monocytes presented as less inflammatory following liraglutide treatment in vivo. This is in keeping with a recent study which showed that lixisenatide decreases proinflammatory monocytes in insulin resistant ApoE^−/−^ mice [35]. Importantly we also showed a significant increase in alternative MΦ2 macrophage populations. This novel atheroprotective effect was further confirmed by analyzing MΦ2 gene expression in BMDMs from liraglutide-treated ApoE^−/−^ mice which showed a statistically significant increase in Arg-1, IL-10, MR and CD163. We have previously shown that induction of IL-10 by MΦ2 macrophages is required for systemic alteration of monocyte populations [50]. This is important in the context of our other work which shows that MΦ2 macrophage accumulation in human atherosclerotic plaque is inversely related to disease progression [13]. Here, we show that as well as enrichment of the MΦ2 phenotype there was a significant reduction in MΦ1 phenotypic markers specifically CCR7 and TNF-alpha.

## Conclusions

In summary our work shows liraglutide has a direct effect on atherosclerosis lesion formation via skewing the macrophage populations towards a pro-resolving MΦ2 phenotype. The effects of liraglutide altering monocyte and macrophage represent a novel mechanism for its atheroprotective effects.

## Additional files

**Additional file 1.** Additional methods and tables.

**Additional file 2: Figure S1.** STAT1 and STAT3 expression in polarized THP-1 macrophages treated with liraglutide. THP-1 monocytes were differentiated into macrophages over 3 days with 320 nM PMA. Cells were rested for 24h in complete medium and polarized into M1 (100 ng/ml LPS and 20 ng/ml IFN-γ) and M2 (20 ng/ml IL-4 and IL-13) macrophages for 48h. Macrophages were treated with 1μg/ml (~250 nM) liraglutide for 6h and protein was taken. The membranes were probed for anti-STAT3, anti-STAT1 and anti-phospho-STAT1 (p-STAT1) all diluted 1:1000 in 5% non-fat milk overnight at 4 °C. Secondary antibodies were anti-rabbit (1:2000) in 5% non-fat milk for 1h at room temperature. β-actin (1:500) (anti-mouse 1:1000) was used as a loading control. Membrane was developed in Super Signal West Pico ECL solution from 1 sec–5 min.

**Additional file 3: Figure S2.** BMDM polarization. WT bones were flushed, cultured in 25% L929-conditioned medium for 7 days and polarized into MΦ1 and MΦ2 macrophages for 18h. a| TNF-alpha and b| MR were analyzed by RT-qPCR. Error bars are representative a| 5 mice (n=5) or b| 3 mice (n=3), each carried out with two replicates. Statistical analysis was performed comparing specific columns using a Wilcoxin-matched pairs signed rank t test. *p<0.05 and **p<0.01 were considered statistically significant. Stars above the columns represent comparisons made against the MΦ0 control.

**Additional file 4: Figure S3.** Quantification of atherosclerotic lesions in aortae of HFHCD-fed ApoE^−/−^ mice. Aortae were harvested from ApoE^−/−^ mice fed a HFHCD for 4–8 weeks. Aortae were harvested and *en face* staining was performed with a| representative images of total aorta lesion areas and percentage lesion quantified for b| total area, c| aortic arch, d| thoracic aorta, e| abdominal aorta and f| iliac bifurcation, using ImageJ. Error bars are representative of 10 aortae per group (n=10). Statistical analysis was performed carrying out Mann−Whitney t tests. Statistical significance was considered when ***p<0.001 and p>0.05 was considered NS.

**Additional file 5: Figure S4.** % weight gain in ApoE^−/−^ mice. ApoE^−/−^ mice were fed a LFD or HFHCD for 2 weeks. From weeks 2–8 mice continued on the diets while also receiving daily subcutaneous injections of 300 μg/kg liraglutide or PBS. Mice were weighed weekly from weeks 1–2 and daily from weeks 2–8. a| represents weights graphed overtime b| incremental weight gain overtime c| % weights of mice during liraglutide dosing period and d| the total % weights of mice for the whole study. Error bars are representative of 16 mice per group (n=16). Statistical analysis was carried out performing a and b| a two-way ANOVA or c and d| a Kruskal-Wallis test followed by Dunn’s multiple comparison post-test. Statistical significance was considered when *p<0.05, **p<0.01 and ***p<0.001. Stars above the columns represent comparisons against the LFD group while capped ines indicate comparisons against other groups.

**Additional file 6: Figure S5.** Plasma cholesterol of ApoE^−/−^ mice. ApoE^−/−^ mice were fed a LFD or HFHCD from weeks 1–8 and received daily injections of 300μg/kg liraglutide (Lir) or PBS from weeks 2–8. Blood samples were taken via retro-orbital plexus. From the plasma and total cholesterol concentration was measured for each group. Error bars are representative of 7 mice per group (n=7). Statistical analysis was performed carrying out a Kruskal–Wallis test followed by Dunn’s multiple comparison post-test. Capped lines indicate comparsions made between groups.

**Additional file 7: Figure S6.** Food and calorie intake of ApoE^−/−^ mice. ApoE^−/−^ mice were fed a LFD or HFHCD for 2 weeks. From weeks 2–8 mice continued on the diets while also receiving daily subcutaneous injections of 300 μg/kg liraglutide (Lir) or PBS. Food intake was measured weekly from weeks 1–8. a and b| represent food intake in grams and c and d| calorie intake over the a and c| the titration period of liraglutide dosing and b and d| for the whole study. Error bars are representative of 4 cages per group (n=4). Statistical analysis was carried out performing a Kruskal–Wallis test followed by Dunn’s multiple comparison post-test. Statistical significance was considered when *p<0.05 and p>0.05 was considered NS. Stars above the columns represent comparisons made against the LFD group.

**Additional file 8: Figure S7.** Water intake of ApoE^-/-^ mice. ApoE^-/-^ mice were fed a LFD or HFHCD for 2 weeks. From weeks 2–8 mice continued on the diets while also receiving daily subcutaneous injections of 300 μg/kg liraglutide or PBS. Water intake was measured weekly from weeks 1–2 and daily from weeks 2–8. a| represents water intake over the titration period of liraglutide dosing and b| for the whole study. Error bars are representative of 4 cages per group (n=4). Statistical analysis was carried out performing a Kruskal–Wallis test followed by Dunn’s multiple comparison post-test. Statistical significance was considered when *p<0.05 and p>0.05 was considered NS.

**Additional file 9: Figure S8.** Glucose measurements from ApoE^-/-^ mice. ApoE^-/-^ mice were fed a LFD or HFHCD for 2 weeks. From weeks 2-8 mice continued on the diets while also receiving daily subcutaneous injections of 300μg/kg liraglutide or PBS. Mice underwent glucose testing every 2 weeks via a tail-vein pin prick procedure. a| represents glucose levels during the liraglutide dosing period (week 2) and b| the glucose levels of mice for the whole study weeks 2-8. Error bars are representative of 16 mice per group (n=16). Statistical analysis was carried out performing a Kruskal–Wallis test followed by Dunn’s multiple comparison post-test. Statistical significance was considered when **p<0.01 while p>0.05 was considered NS. Stars above the boxes represent comparisons against the LFD group.

**Additional file 10: Figure S9.** Gating strategy for MΦ1 and MΦ2 markers analysing bone marrow-derived monocytes and macrophages. Monocytes (suspension) and macrophage (adherent) populations from BMDMs were selected and analyzed using the above flow cytometry antibodies in the above sequence. %’s were calculated from the final populations against the total number of cells acquired.

**Additional file 11: Figure S10.** Macrophage EAT infiltration with *in vivo* liraglutide treatment. Adipose tissue macrophages were extracted from the epididymal adipose tissue and stained with the above antibodies and analyzed by flow cytometry in the above sequence. % macrophages were calculated based on total number of cells acquired and final macrophage numbers in a| total macrophage infiltration and b| MΦ1 and MΦ2 EAT macrophages. Error bars are representative of a minimum of 8 mice per group (n=8). Statistical analysis was carried out performing Kruskal-Wallis tests followed by Dunn’s multiple comparison post-tests. p>0.05 was considered NS. Capped lines represent comparisons made between groups.

## Abbreviations

ApoE^−/−^: apolipoprotein E knockout
Arg-1: arginase-1
BMDMs: bone marrow-derived macrophages
CCR7: C-C motif chemokine receptor 7
CVD: cardiovascular disease
DM: diabetes mellitus
EAT: epididymal adipose tissue
GLP-1R: glucagon-like peptide-1 receptor
HFHCD: high-fat (60%) high-cholesterol (1%) diet
iNOS: inducible nitric oxide synthase
LEADER trial: liraglutide effect and action in diabetes: evaluation of cardiovascular outcome results trial
LFD: low-fat diet
LPS: lipopolysaccharide
MCP-1: monocyte chemoattractant protein-1
MR: mannose receptor C-type 1
PBMCs: peripheral blood mononuclear cells
Phospho: p
PMA: phorbol 12-myristate 13-acetate
RT-qPCR: reverse transcription-qPCR
STAT: signal and activator of transcription
WT: wild-type C57BL/6
