# Supplementary material for: Liraglutide dictates macrophage phenotype in apolipoprotein E null mice during early atherosclerosis
Source: Cardiovasc Diabetol. 2017 Nov 6;16:143. doi: 10.1186/s12933-017-0626-3 (PMC5674826; doi:10.1186/s12933-017-0626-3)
Supplement: Supplementary file 1 — Additional file 1. Additional methods and tables. [file 12933_2017_626_MOESM1_ESM.docx]

**Electronic Supplementary Material (ESM)**

**Additional Abbreviations**

| SVF | Stromal vascular fraction |
| --- | --- |
| SDS | Sodium dodecyl sulfate |

**Additional Methods**

**ApoE^-/-^ experimental number justification**

The study animals will be inbred mice on a uniform ApoE^-/-^ genetic background. This helps to minimize inter-animal variability and thus reduce animal numbers. The number of mice has been reduced in each experiment to the absolute minimum to achieve statistical significance once we factor in significant physiologic variability in the animals. Further reduction in the number of animals requested per experiment would result in uninterpretable data. A pilot study performed at the University of Pennslyvania demonstrated ~15% variance between control and obese animals in plasma 3H-cholesterol counts at 4h during RCT; μ1=0.458; μ2=0.389 and pooled variance σ=0.07. We estimate that to achieve 80% power (beta, β) with a probability of 0.05 (alpha, α) or less of false positives or negatives, the number of animals required per group is 16 animals per group.

From the normal tables zα/2 =Z 0.025 = 1.96 and Zβ = Z0.20 = 0.842

N=2σ2(zα/2 + zβ)2 / (μ1 – μ2)2

N= 2(0.07) 2 (1.96+0.842)2/ (0.458-0.389)2

N=(0.0757/0.004761)

N=(15.9)

=16

**Plasma cholesterol analysis**

Plasma cholesterol concentrations were quantified enzymatically using LabAssay^TM^ Cholesterol (Wako Chemicals GmbH, Neuss, Germany) as per the Manufacturer’s instructions.

**EAT macrophage processing and flow cytometry labelling**

To separate the stromal vascular fraction (SVF) from adipocytes, EAT was minced and then collagenase (2mg/ml) (Invitrogen, Thermo Fisher Scientific, Waltham, MA, USA) digested for 60min. Adipocytes were removed and digested EAT suspension was filtered and centrifuged for 5min at 1,700 rpm. The SVF was re-suspended and blocked in 2% BSA/PBS. Cells were stained with antibodies (ESM Table 3) for 30min on ice in the dark, washed and re-suspended in 2%BSA/PBS before running on the BD Accuri C6 Cytometer (BD Bioscience, Oxford, UK). Amine C reactive beads and cells were stained with antibodies to generate single stained controls and fluorescence minus one controls for gating and compensation. FCS files were analyzed using FlowLogic software (Miltenyi Biotec Ltd., Surrey, UK).

**Additional Tables**

Table S1 – 10% sodium dodecyl sulfate (SDS)-polyacrylamide gel electrophoresis

| **Reagent** | **1 Gel** |
| --- | --- |
| **Resolving Gel*** | |
| Deionized water | 4ml |
| 30% acrylamide Mix | 3.3ml |
| 1.5mol/l tris (pH 8.8) | 2.5ml |
| 10% SDS | 100μl |
| 10% ammonium persulfate | 100μl |
| Tetramethylethylenediamine | 4μl |
| **Stacking Gel*** | |
| Deionized water | 5.5ml |
| 30% acrylamide Mix | 1.3ml |
| 1mol/l tris (pH 6.8) | 1ml |
| 10% SDS | 80μl |
| 10% ammonium persulfate | 80μl |
| Tetramethylethylenediamine | 8μl |

*All reagents were bought from Sigma Aldrich, Wicklow, Ireland.

Table S2 – SYBR® Green primer sequences

| Gene Name | Gene Sequence | HGNC ID |
| --- | --- | --- |
| *Arg1* forward | 5’-CAGAAGAATGGAAGAGTCAG-3’ | 663 |
| *Arg1* reverse | 5’-CAGATATGCAGGGAGTCACC-3’ | 663 |
| *CD163* forward | 5’-CGAGTTAACGCCAGTAAGG-3’ | 1631 |
| *CD163* reverse | 5’-GAACATGTCACGCCAGC-3’ | 1631 |
| *MCP1* forward | 5’-CTGCTCATAGCAGCCACCTT-3’ | 10618 |
| *MCP1* reverse | 5’-CAGGTGACTGGGGCATTG-3’ | 10618 |
| *Mrc1* forward | 5’-ATGCCAAGTGGGAAAATCTG-3’ | 7228 |
| *Mrc1* reverse | 5’-TGTAGCAGTGGCCTGCATAG-3’ | 7228 |
| *TNF-alpha* forward | 5’-CTCGAACCCCGAGTGACAA-3’ | 11894 |
| *TNF-alpha* reverse | 5’-GCTGCCCCTCAGCTTGAG-3’ | 11894 |

Table S3 – Antibodies for EAT flow cytometry

| Marker | Flurochrome | Isotype | Clone | Supplier |
| --- | --- | --- | --- | --- |
| F4/80 | Fluorescein isothiocyanate (FITC) | rat IgG2b, κ | BM8 | eBioscience, San Diego, CA, USA. |
| CD11c | R-PE | hamster IgG1, λ1 | N418 | ABD Serotec, Kidlington, UK |
| CD206 | Alexa Fluor®647/PE | rat IgG2a | MR5D3 | ABD Serotec Kidlington, UK |
| CD11b | Peridinin Chlorophyll Protein Complex-Cyanine 5.5 (PerCP-Cy™5.5) | rat IgG_2b_, κ | M1/70 | BD Bioscience, Oxford, UK |
