# Supplementary figures and images for: Liraglutide dictates macrophage phenotype in apolipoprotein E null mice during early atherosclerosis
Source: Cardiovasc Diabetol. 2017 Nov 6;16:143. doi: 10.1186/s12933-017-0626-3 (PMC5674826; doi:10.1186/s12933-017-0626-3)

## Slide 1
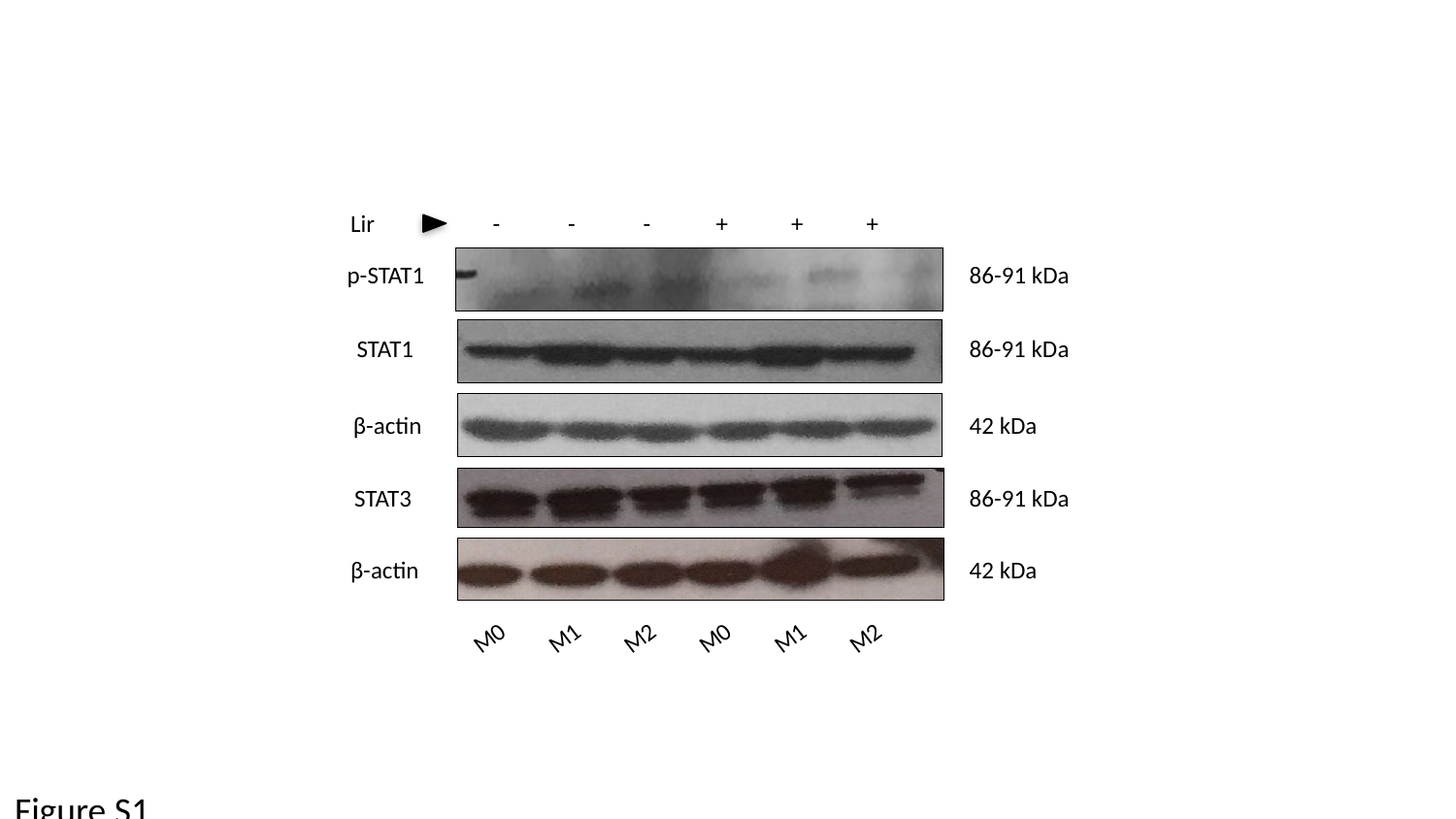

Lir
-
-
-
+
+
+
p-STAT1
86-91 kDa
β-actin
42 kDa
STAT3
86-91 kDa
β-actin
42 kDa
M0
M1
M2
M0
M1
M2
STAT1
86-91 kDa
Figure S1

Supplement: Supplementary file 2 — Additional file 2: Figure S1. STAT1 and STAT3 expression in polarized THP-1 macrophages treated with liraglutide. THP-1 monocytes were differentiated into macrophages over 3 days with 320 nM PMA. Cells were rested for 24h in complete medium and polarized into M1 (100 ng/ml LPS and 20 ng/ml IFN-γ) and M2 (20 ng/ml IL-4 and IL-13) macrophages for 48h. Macrophages were treated with 1μg/ml (~250 nM) liraglutide for 6h and protein was taken. The membranes were probed for anti-STAT3, anti-STAT1 and anti-phospho-STAT1 (p-STAT1) all diluted 1:1000 in 5% non-fat milk overnight at 4 °C. Secondary antibodies were anti-rabbit (1:2000) in 5% non-fat milk for 1h at room temperature. β-actin (1:500) (anti-mouse 1:1000) was used as a loading control. Membrane was developed in Super Signal West Pico ECL solution from 1 sec–5 min. [file 12933_2017_626_MOESM2_ESM.pptx]

## Slide 1
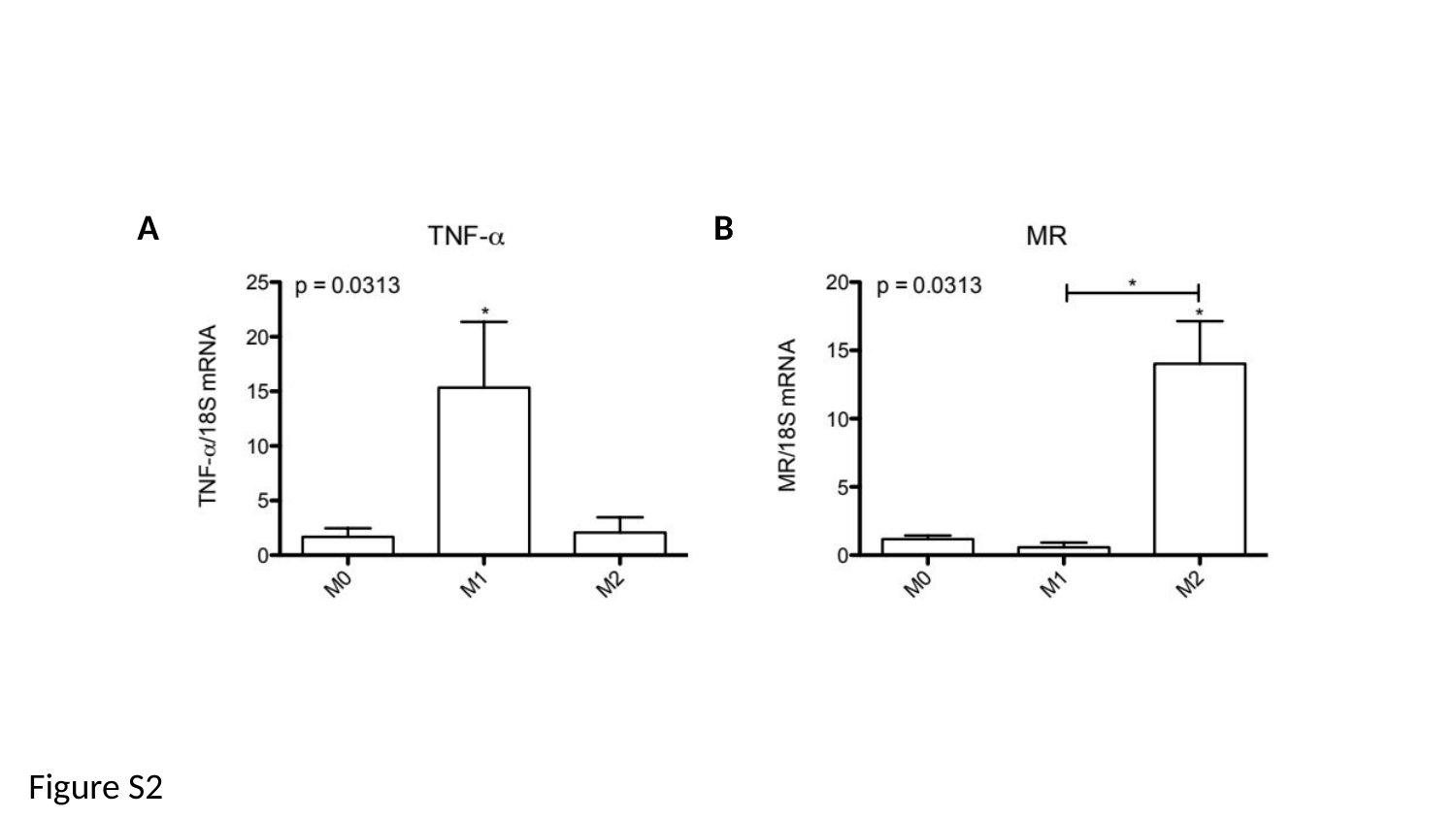

A
B
Figure S2

Supplement: Supplementary file 3 — Additional file 3: Figure S2. BMDM polarization. WT bones were flushed, cultured in 25% L929-conditioned medium for 7 days and polarized into MΦ1 and MΦ2 macrophages for 18h. a| TNF-alpha and b| MR were analyzed by RT-qPCR. Error bars are representative a| 5 mice (n=5) or b| 3 mice (n=3), each carried out with two replicates. Statistical analysis was performed comparing specific columns using a Wilcoxin-matched pairs signed rank t test. *p<0.05 and **p<0.01 were considered statistically significant. Stars above the columns represent comparisons made against the MΦ0 control. [file 12933_2017_626_MOESM3_ESM.pptx]

## Slide 1
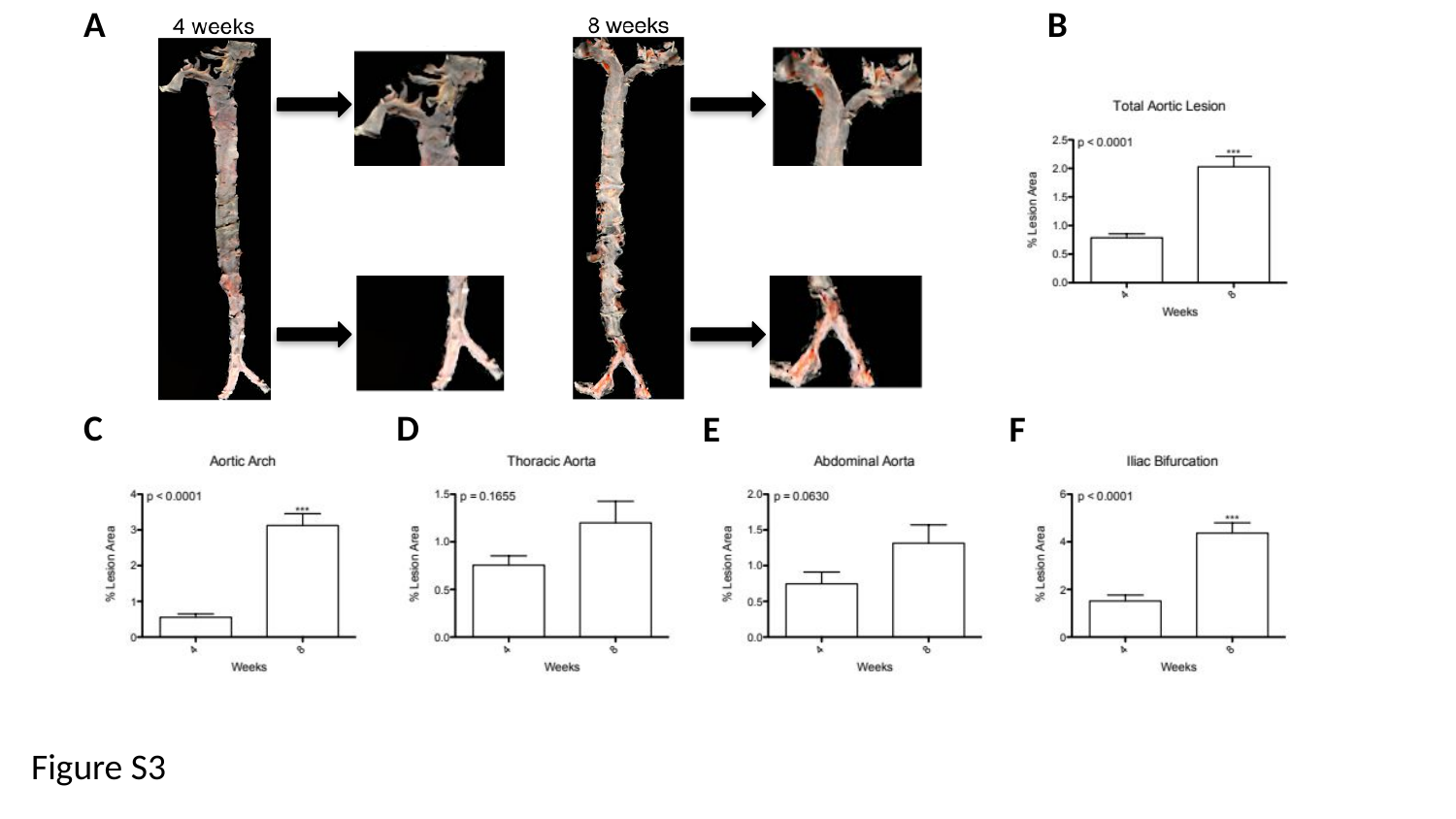

A
B
C
D
E
F
Figure S3

Supplement: Supplementary file 4 — Additional file 4: Figure S3. Quantification of atherosclerotic lesions in aortae of HFHCD-fed ApoE−/− mice. Aortae were harvested from ApoE−/− mice fed a HFHCD for 4–8 weeks. Aortae were harvested and en face staining was performed with a| representative images of total aorta lesion areas and percentage lesion quantified for b| total area, c| aortic arch, d| thoracic aorta, e| abdominal aorta and f| iliac bifurcation, using ImageJ. Error bars are representative of 10 aortae per group (n=10). Statistical analysis was performed carrying out Mann−Whitney t tests. Statistical significance was considered when ***p<0.001 and p>0.05 was considered NS. [file 12933_2017_626_MOESM4_ESM.pptx]

## Slide 1
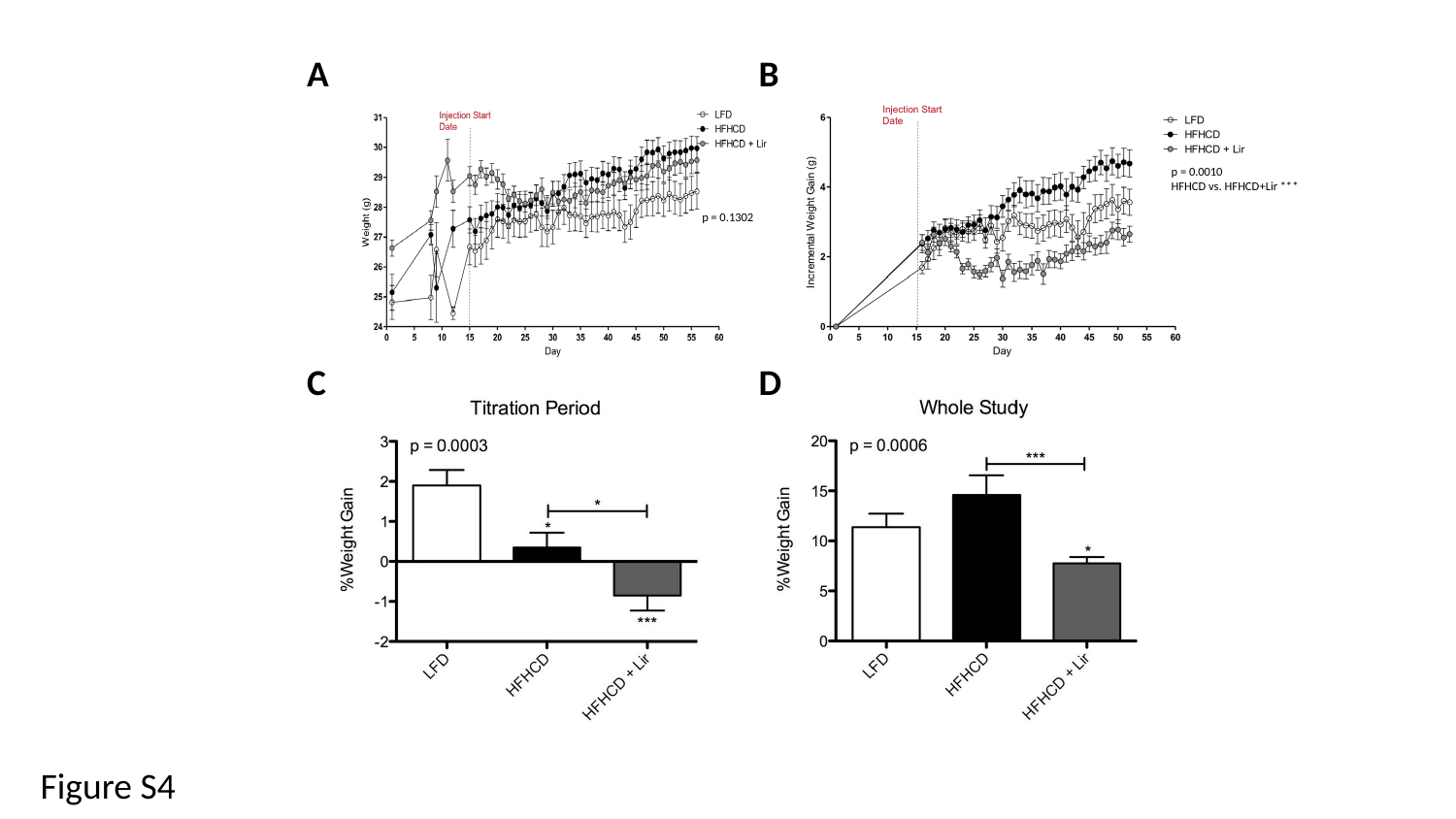

A
B
p = 0.0010
HFHCD vs. HFHCD+Lir ***
p = 0.1302
C
D
Figure S4

Supplement: Supplementary file 5 — Additional file 5: Figure S4. % weight gain in ApoE−/− mice. ApoE−/− mice were fed a LFD or HFHCD for 2 weeks. From weeks 2–8 mice continued on the diets while also receiving daily subcutaneous injections of 300 μg/kg liraglutide or PBS. Mice were weighed weekly from weeks 1–2 and daily from weeks 2–8. a| represents weights graphed overtime b| incremental weight gain overtime c| % weights of mice during liraglutide dosing period and d| the total % weights of mice for the whole study. Error bars are representative of 16 mice per group (n=16). Statistical analysis was carried out performing a and b| a two-way ANOVA or c and d| a Kruskal-Wallis test followed by Dunn’s multiple comparison post-test. Statistical significance was considered when *p<0.05, **p<0.01 and ***p<0.001. Stars above the columns represent comparisons against the LFD group while capped ines indicate comparisons against other groups. [file 12933_2017_626_MOESM5_ESM.pptx]

## Slide 1
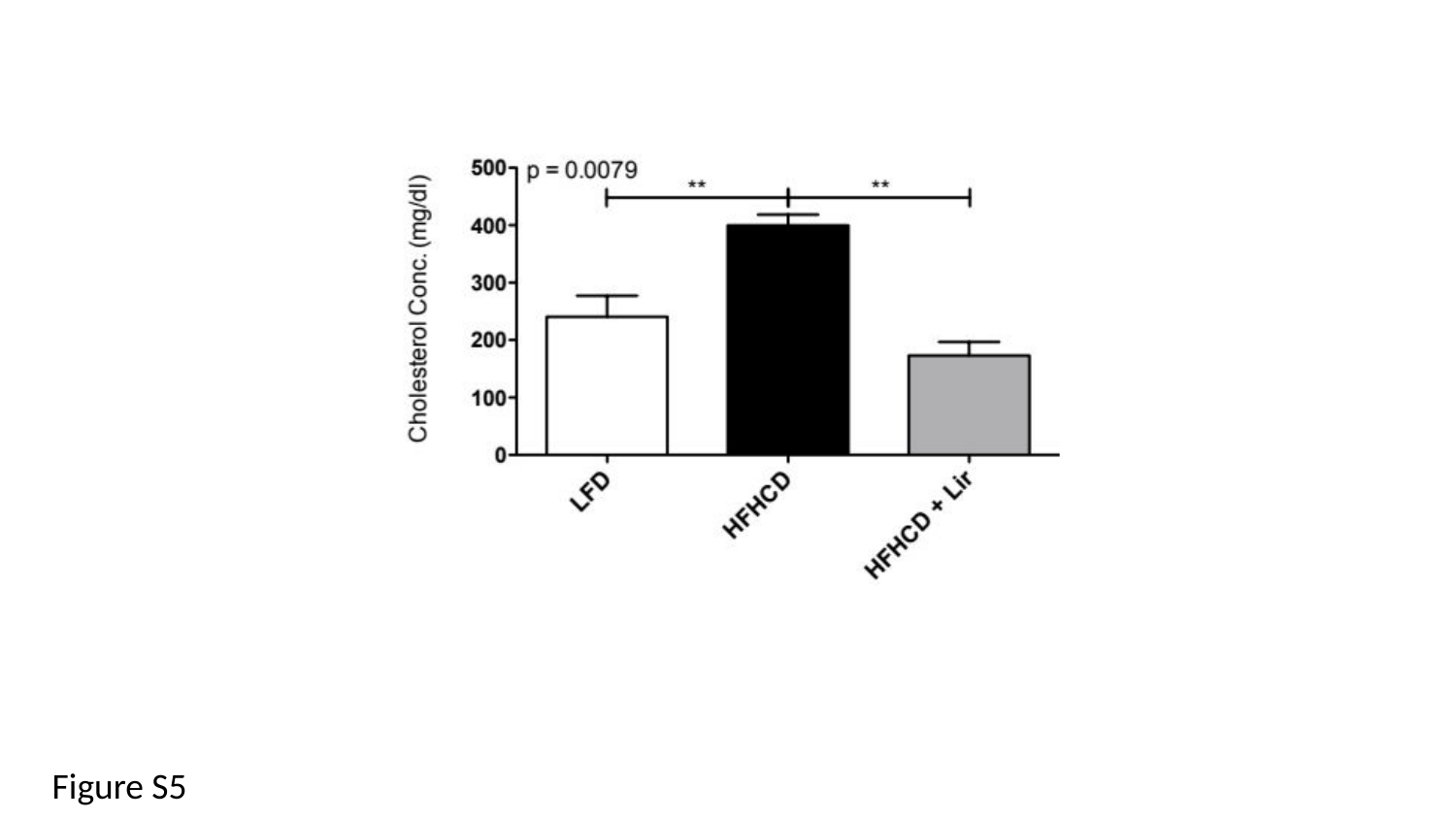

Figure S5

Supplement: Supplementary file 6 — Additional file 6: Figure S5. Plasma cholesterol of ApoE−/− mice. ApoE−/− mice were fed a LFD or HFHCD from weeks 1–8 and received daily injections of 300μg/kg liraglutide (Lir) or PBS from weeks 2–8. Blood samples were taken via retro-orbital plexus. From the plasma and total cholesterol concentration was measured for each group. Error bars are representative of 7 mice per group (n=7). Statistical analysis was performed carrying out a Kruskal–Wallis test followed by Dunn’s multiple comparison post-test. Capped lines indicate comparsions made between groups. [file 12933_2017_626_MOESM6_ESM.pptx]

## Slide 1
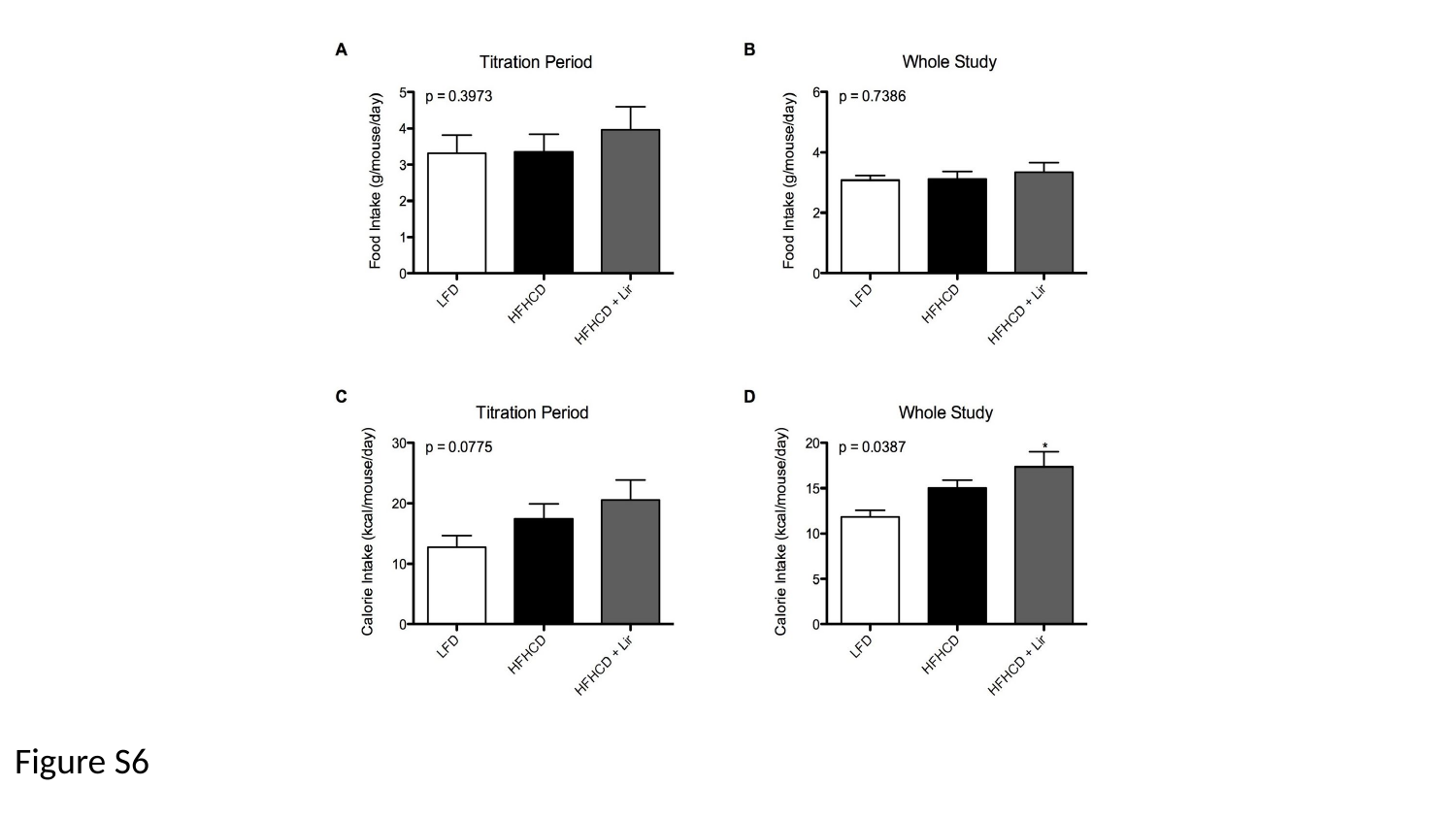

Figure S6

Supplement: Supplementary file 7 — Additional file 7: Figure S6. Food and calorie intake of ApoE−/− mice. ApoE−/− mice were fed a LFD or HFHCD for 2 weeks. From weeks 2–8 mice continued on the diets while also receiving daily subcutaneous injections of 300 μg/kg liraglutide (Lir) or PBS. Food intake was measured weekly from weeks 1–8. a and b| represent food intake in grams and c and d| calorie intake over the a and c| the titration period of liraglutide dosing and b and d| for the whole study. Error bars are representative of 4 cages per group (n=4). Statistical analysis was carried out performing a Kruskal–Wallis test followed by Dunn’s multiple comparison post-test. Statistical significance was considered when *p<0.05 and p>0.05 was considered NS. Stars above the columns represent comparisons made against the LFD group. [file 12933_2017_626_MOESM7_ESM.pptx]

## Slide 1
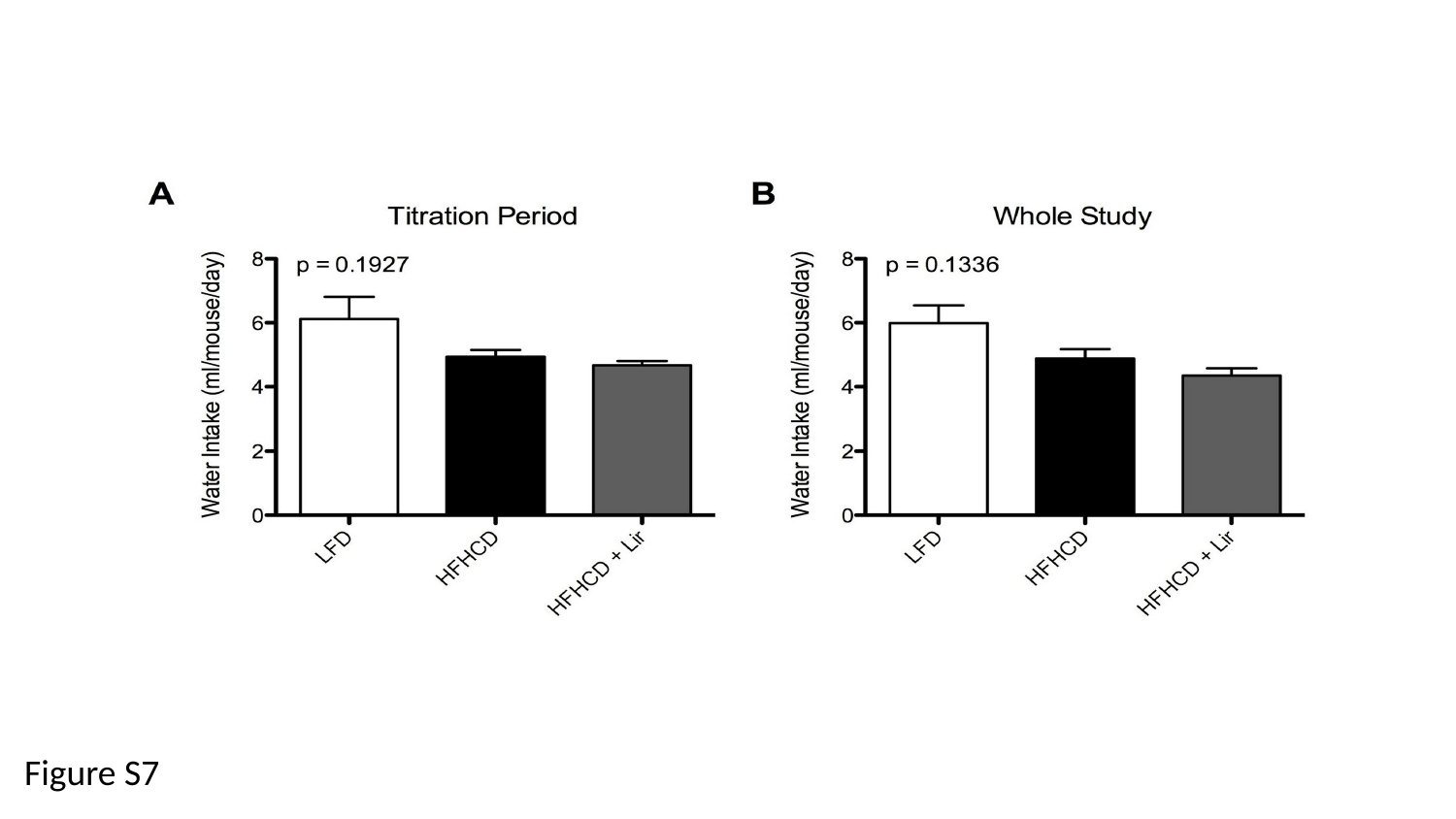

Figure S7

Supplement: Supplementary file 8 — Additional file 8: Figure S7. Water intake of ApoE-/- mice. ApoE-/- mice were fed a LFD or HFHCD for 2 weeks. From weeks 2–8 mice continued on the diets while also receiving daily subcutaneous injections of 300 μg/kg liraglutide or PBS. Water intake was measured weekly from weeks 1–2 and daily from weeks 2–8. a| represents water intake over the titration period of liraglutide dosing and b| for the whole study. Error bars are representative of 4 cages per group (n=4). Statistical analysis was carried out performing a Kruskal–Wallis test followed by Dunn’s multiple comparison post-test. Statistical significance was considered when *p<0.05 and p>0.05 was considered NS. [file 12933_2017_626_MOESM8_ESM.pptx]

## Slide 1
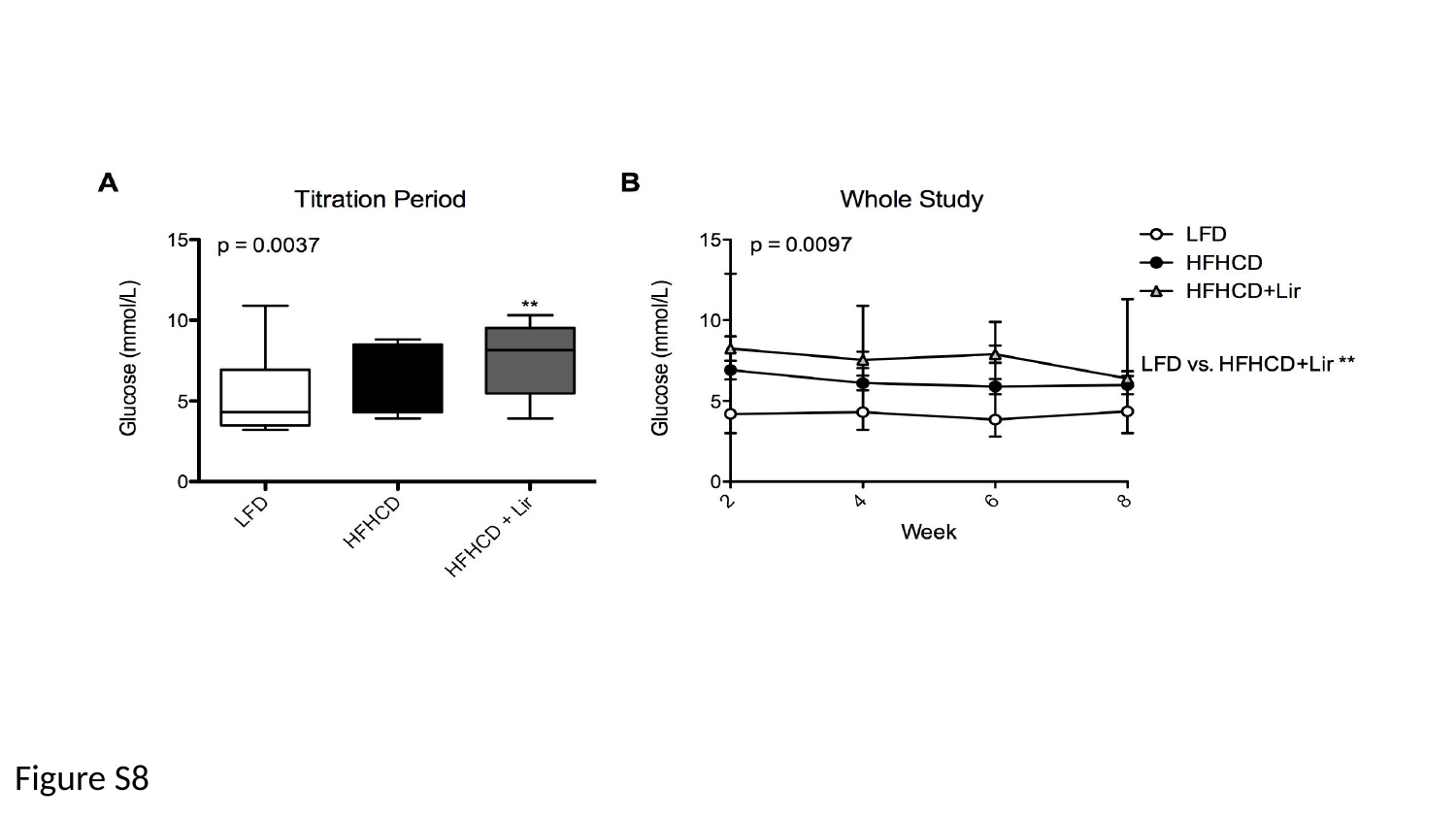

Figure S8

Supplement: Supplementary file 9 — Additional file 9: Figure S8. Glucose measurements from ApoE-/- mice. ApoE-/- mice were fed a LFD or HFHCD for 2 weeks. From weeks 2-8 mice continued on the diets while also receiving daily subcutaneous injections of 300μg/kg liraglutide or PBS. Mice underwent glucose testing every 2 weeks via a tail-vein pin prick procedure. a| represents glucose levels during the liraglutide dosing period (week 2) and b| the glucose levels of mice for the whole study weeks 2-8. Error bars are representative of 16 mice per group (n=16). Statistical analysis was carried out performing a Kruskal–Wallis test followed by Dunn’s multiple comparison post-test. Statistical significance was considered when **p<0.01 while p>0.05 was considered NS. Stars above the boxes represent comparisons against the LFD group. [file 12933_2017_626_MOESM9_ESM.pptx]

## Slide 1
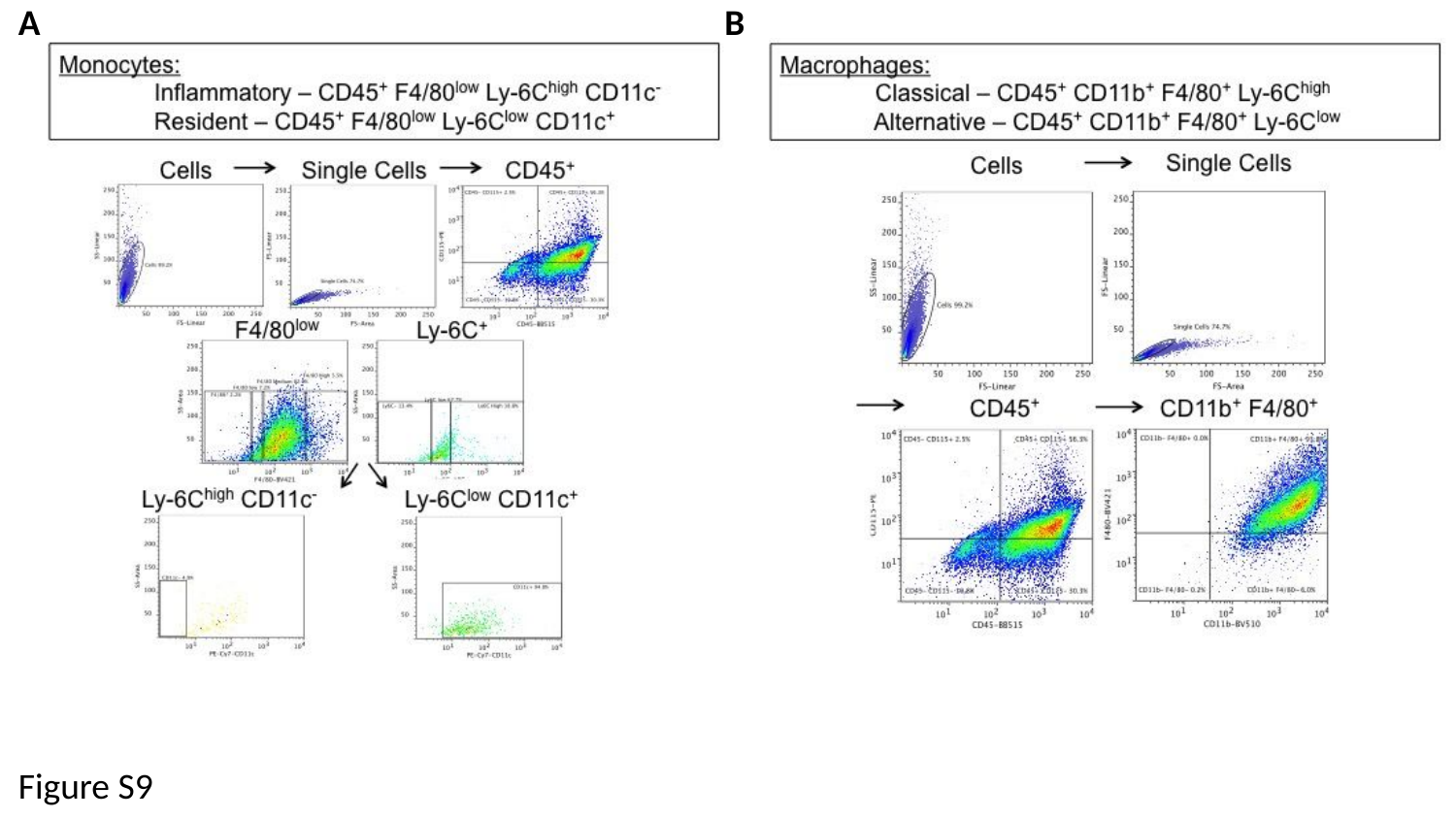

A
B
Figure S9

Supplement: Supplementary file 10 — Additional file 10: Figure S9. Gating strategy for MΦ1 and MΦ2 markers analysing bone marrow-derived monocytes and macrophages. Monocytes (suspension) and macrophage (adherent) populations from BMDMs were selected and analyzed using the above flow cytometry antibodies in the above sequence. %’s were calculated from the final populations against the total number of cells acquired. [file 12933_2017_626_MOESM10_ESM.pptx]

## Slide 1
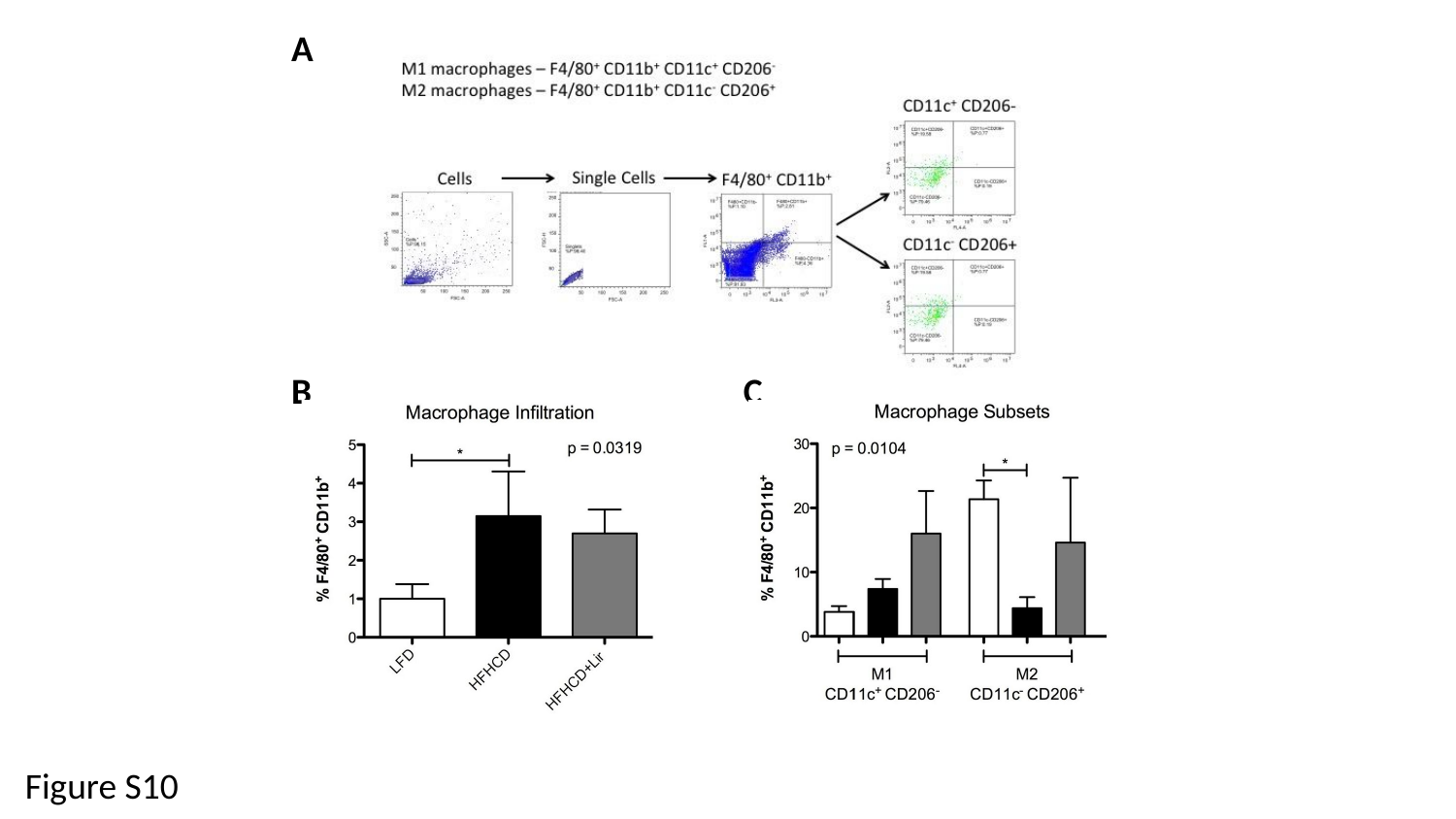

A
B
C
Figure S10

Supplement: Supplementary file 11 — Additional file 11: Figure S10. Macrophage EAT infiltration with in vivo liraglutide treatment. Adipose tissue macrophages were extracted from the epididymal adipose tissue and stained with the above antibodies and analyzed by flow cytometry in the above sequence. % macrophages were calculated based on total number of cells acquired and final macrophage numbers in a| total macrophage infiltration and b| MΦ1 and MΦ2 EAT macrophages. Error bars are representative of a minimum of 8 mice per group (n=8). Statistical analysis was carried out performing Kruskal-Wallis tests followed by Dunn’s multiple comparison post-tests. p>0.05 was considered NS. Capped lines represent comparisons made between groups. [file 12933_2017_626_MOESM11_ESM.pptx]
